# Anatomical and Biochemical Changes Induced by *Gluconacetobacter diazotrophicus* Stand Up for *Arabidopsis thaliana* Seedlings From *Ralstonia solanacearum* Infection

**DOI:** 10.3389/fpls.2019.01618

**Published:** 2019-12-23

**Authors:** María V. Rodriguez, Josefina Tano, Nazarena Ansaldi, Analía Carrau, María S. Srebot, Virginia Ferreira, María L. Martínez, Adriana A. Cortadi, María I. Siri, Elena G. Orellano

**Affiliations:** ^1^Área Biología Vegetal (CONICET), Facultad de Ciencias Bioquímicas y Farmacéuticas, Universidad Nacional de Rosario, Rosario, Argentina; ^2^Facultad de Ciencias Bioquímicas y Farmacéuticas, Instituto de Biología Molecular y Celular de Rosario (CONICET-UNR), Universidad Nacional de Rosario, Rosario, Argentina; ^3^Área Biología Molecular (CONICET), Facultad de Ciencias Bioquímicas y Farmacéuticas, Universidad Nacional de Rosario, Rosario, Argentina; ^4^Departamento de Biociencias, Facultad de Química, Universidad de la República, Montevideo, Uruguay

**Keywords:** biocontrol, *Gluconacetobacter diazotrophicus*, induced systemic resistance, plant growth promoting bacteria endophyte, *Ralstonia solanacearum*

## Abstract

Nowadays, fertilization and pest control are carried out using chemical compounds that contaminate soil and deteriorate human health. Plant growth promoting bacteria endophytes (PGPBEs), are a well-studied group of bacteria that offers benefits to the host plant, such as phytostimulation, biofertilization, and protection against other microorganisms. The study of *Gluconacetobacter diazotrophicus*–which belongs to PGPBEs-aids the development of alternative strategies of an integrated approach for crop management practices. *Ralstonia solanacearum* is responsible for bacterial wilt disease. This phytopathogen is of great interest worldwide due to the enormous economic losses it causes. In this study the action of *G. diazotrophicus* as a growth promoting bacterium in *Arabidopsis thaliana* seedlings is analyzed, evaluating the antagonistic mechanisms of this beneficial endophytic bacterium during biotic stress produced by *R. solanacearum*. Effective colonization of *G. diazotrophicus* was determined through bacterial counting assays, evaluation of anatomical and growth parameters, and pigments quantification. Biocontrol assays were carried out with *Ralstonia pseudosolanacearum* GMI1000 model strain and *R. solanacearum* A21 a recently isolated strain. Inoculation of *A. thaliana* (Col 0) with *G. diazotrophicus* Pal 5 triggers a set of biochemical and structural changes in roots, stems, and leaves of seedlings. Discrete callose deposits as papillae were observed at specific sites of root hairs, trichomes, and leaf tissue. Upon *R. pseudosolanacearum* GMI1000 infection, endophyte-treated plants demonstrated being induced for defense through an augmented callose deposition at root hairs and leaves compared with the non-endophyte-treated controls. The endophytic bacterium appears to be able to prime callose response. Roots and stems cross sections showed that integrity of all tissues was preserved in endophyte-treated plants infected with *R. solanacearum* A21. The mechanisms of resistance elicited by the plant after inoculation with the endophyte would be greater lignification and sclerosis in tissues and reinforcement of the cell wall through the deposition of callose. As a consequence of this priming in plant defense response, viable phytopathogenic bacteria counting were considerably fewer in endophyte-inoculated plants than in not-inoculated controls. Our results indicate that *G. diazotrophicus* colonizes *A. thaliana* plants performing a protective role against the phytopathogenic bacterium *R. solanacearum* promoting the activation of plant defense system.

## Introduction

Global climate change and increase in human population generate large pressure over natural resources, including the demand of land and water resources available for food production. In addition, plant diseases represent a serious threat to agricultural crops (Velivelli et al., 2014). Chemical pesticides used for the control of phytopathogens are currently known for their adverse effects both on the environment and on the health of consumers. This issue has become a matter of growing concern among consumers and generates social pressure for food free of pesticide residues (Gerbore et al., 2014). Legislation limiting the use of certain agrichemicals, the high awareness, and the lack of acceptance by consumers of genetically modified crops, in addition to their strict regulation, leads the development of new sustainable practices for agriculture. The immediate task that stakeholders face is the search for a sustainable crop production system to solve the problems that threaten global food security. An example of such a sustainable crop-production system is the strategy that incorporates beneficial microorganisms to improve plant health.

The beneficial effects of bacterial endophytes on host plants appear to take place through two types of mechanisms: direct growth promotion activity or indirect mechanisms (Velivelli et al., 2014). Within the direct activity of growth promotion there are several ways in which different PGPB directly facilitate the proliferation of their host plants: they can fix atmospheric nitrogen and supply it to plants, synthesize different phytohormones to intensify the growth of the host plant; they also have solubilization mechanisms of minerals such as phosphorus, to improve their availability. A particular endophytic microorganism can affect the growth of the plant and its development through the use of one or more of these mechanisms. Endophytic bacteria reduce or prevent the deleterious effects of phytopathogenic organisms, and this ability can be considered as an indirect promotion of plant growth (Lodewyckx et al., 2002). The direct inhibition of pathogens carried out by endophytic bacteria is commonly mediated by the synthesis of inhibitory allelochemicals such as antibiotics, iron chelating siderophores, antifungal metabolites, and the degradation of signals produced by pathogens (quorum sensing quenching). Indirect biocontrol mechanisms of endophytic bacteria include the induction of systemic resistance in plants that inhibits a broad spectrum of phytopathogens (Liu et al., 2017). Disease elimination by the biocontrol agents is the prolonged manifestation of the interaction among the plant, the phytopathogen, the biocontrol agent, the microbial community surrounding the plant and the environment (Handelsman and Stabb, 1996).

The induced state of systemic resistance (ISR) is characterized by the activation of latent defense mechanisms that are subsequently expressed more rapidly and intensively in response to an infection by a pathogen at a low physiological cost for the plant. The ISR is characterized for being activated after the interaction between beneficial microorganisms and their host plants; this induction is signaled by the ethylene (ET) and jasmonic acid (JA) or salicylic acid (SA) pathways or a combination of both signaling pathways. When the induced resistance is demonstrated to be SA dependent, is referred to as systemic acquired resistance (SAR) (Pieterse et al., 2014). Several studies showed that relatively slight changes occurred in the transcriptome in systemic tissues upon a colonization of the roots by a beneficial microorganism, especially when compared with the massive transcriptional reprogramming that occurs during the attack of pathogens (Verhagen et al., 2004). Because the defense mechanisms remain dormant after interaction with beneficial microorganisms, it is sometimes difficult to recognize them in plants that have not been challenged by an interaction with a pathogen; therefore the combination in the interaction plant-beneficial microorganism-pathogen allows studying and visualizing the ISR changes easily (Pieterse et al., 2014).

*Gluconacetobacter diazotrophicus* is a Gram-negative bacterium, tolerant to acid, obligate aerobic and rod-shaped with rounded ends (0.7–0.9 µm x 1–2 µm) with lateral or peritrichous flagella (Cavalcante and Dobereiner, 1988; Gillis et al., 1989; Muthukumarasamy et al., 2002; Chawla et al., 2014). *Gluconacetobacter* belongs to the *Proteobacteria* phylum, in the α-proteobacteria section, *Rhodospirillales* order and *Acetobacteraceae* family (Kersters et al., 2006). *G. diazotrophicus* (Yamada et al., 1997) formerly named *Acetobacter diazotrophicus*, (Gillis et al., 1989). This bacterium was originally isolated from sugar cane (Cavalcante and Dobereiner, 1988) and has the ability to fix atmospheric nitrogen without forming nodules (Stephan et al., 1991; Alvarez and Martínez-Drets, 1995). Its endophytic nature was confirmed in Brazil by the counting of this bacterium in roots, stems, and aerial parts of sugarcane (Reis et al., 1994). The potentially beneficial effects promoted by this bacterium in plants are nitrogen fixation, phytohormones production, inhibition/suppression of pathogen, and solubilization of mineral nutrients (Fuentes-Ramirez et al., 1993; Fisher and Newton, 2005). In 2009, the genome of the Pal5 strain of *G. diazotrophicus* was completely sequenced and genes involved in nitrogen fixation, sugar metabolism, transport systems, polysaccharide biosynthesis, quorum sensing, and auxin biosynthesis were identified, confirming its importance (Bertalan et al., 2009). The potential use of *G. diazotrophicus* as antagonist against *Colletotrichum falcatum*, the pathogenic fungus of “red rot” in sugarcane was first demonstrated by Muthukumarasamy et al. (2000). When *G. diazotrophicus* and the fungal pathogen were cultured in the same medium, a clear zone of inhibition against the pathogen was visualized. Similarly, its potential as antagonist of *Xanthomonas albilineans*, the organism responsible for leaf scald disease in sugarcane, was demonstrated. *G. diazotrophicus* secretes certain proteins (bacteriocins) that prevent the growth of *X. albilineans* and imparts a lysozyme-like activity to the inner cell wall of the pathogen (Blanco et al., 2005). *G. diazotrophicus* presented antifungal activity against several species of *Fusarium spp*. when they were grown on potato-dextrose agar (PDA) (Logeshwarn et al., 2011). Regarding the induction of resistance in the host plant by *G. diazotrophicus*, there are reports that show an increase in a marker of the JA/ET defense pathway in rice plants when these were inoculated with this endophytic bacterium (Filgueiras et al., 2019). The activation of genes involved in the ET signaling pathway in sugarcane plants colonized by *G. diazotrophicus* was also demonstrated (De Nogueira et al., 2001; Cavalcante et al., 2007). In addition, the accumulation of polysaccharides and tannins in the parenchymal cells surrounding the metaxylem of sugarcane plants inoculated with *G. diazotrophicus* was reported, suggesting that the plant’s defense system is activated during the interaction with the bacterium (Dong et al., 1997). The inoculation of *G. diazotrophicus* in plants of *A. thaliana*, *NahG*, mutated in the SAR, revealed that this route of signaling related to the immune system of the plant plays an important role during the stages of early association of the endophytic bacterium with the host plant (Rangel de Souza et al., 2015). The activation of these defense systems after *G. diazotrophicus inoculation* indicates their role in the biocontrol of pathogens by priming.

*Ralstonia solanacearum* is a phytopathogenic β-proteobacteria of great importance worldwide due to the enormous economic losses that it causes, since it attacks a wide variety of crops and wild plants (Hayward, 1991; Genin and Denny, 2012; Peeters et al., 2013). *R. solanacearum* attacks more than 200 species of plants belonging to more than 60 different botanical families, affecting not only solanaceous plants such as potato and tomato but also many agricultural crops, shrubs, trees, and weeds. This unusual wide host range expands continuously, and descriptions of new hosts are very common. This bacterium produces the disease known as bacterial wilt, which is characterized by the loss of leaf turgor and general decay of the whole plant, due to the obstruction of the conducting tissues that transport water and nutrients throughout the stem (Gabor and Wiebe, 1997). Although it is generally considered a plant pathogen, *R. solanacearum* mainly behaves like a saprophyte bacterium capable of surviving for long periods of time in various natural habitats, such as superficial water and different types of soil. As a consequence, it is able to use the wide variety of carbon sources and face the toxic compounds present in the soil. The bacterium has a large repertoire of catabolic genes, genes responsible for the detoxification of harmful compounds and adhesion, which allow efficient colonization and permanence in specific ecological niches (Genin and Boucher, 2004).

This work investigates the action of *G. diazotrophicus* as a growth promoting bacterium in *A. thaliana* seedlings, evaluating the antagonistic mechanisms of this beneficial endophytic bacterium during the biotic stress produced by *Ralstonia pseudosolanacearum* GMI1000 and *R. solanacearum* A21. These strains attack agronomic crops of interest worldwide. In addition, in this work we have genotyped the strains of *R. solanacearum* isolated from the Northeastern region of Argentina.

## Materials and Methods

### Bacterial Strains and Growth Conditions

*G. diazotrophicus* Pal 5 was kindly ceded by Ing. Agr. Paola Delaporte Quintana, who works in the Instituto Superior de Investigaciones Biológicas (INSIBIO, Tucumán, Argentina). *R. solanacearum* A21, phylotype IIA-sequevar 50 strain isolated from tomato in Argentina, was obtained from Culture Collection of the Intituto Nacional de Tecnología Agropecuaria (INTA Bella Vista, Corrientes, Argentina) and typified by Dr. MI Siri and Bioq. V Ferreira (Departamento de Biociencias, Facultad de Química, Universidad de la República, Montevideo, Uruguay). In the present work, the strain *R. pseudosolanacearum* GMI1000, wild reference strain (Salanoubat et al., 2002; Prior et al., 2016; Safni et al., 2014) was also used. *R. pseudosolanacearum* reporter strain that express the green fluorescence protein (GFP) constitutively, GMI1000-GFP was constructed, validated previously, and kindly provided by Dr. Marc Valls (CRAG, Barcelona, España;Monteiro et al., 2012b). The reporter system was introduced in a neutral genome region of *R. pseudosolanacearum* GMI1000 (Monteiro et al., 2012b).

Cells of *G. diazotrophicus* strain Pal 5 were grown in LGI-P medium (g L^−1^): sucrose, 100.0; K_2_HPO_4_, 0.2; KH_2_PO_4_, 0.6; MgSO_4_·7H_2_O, 0.2; CaCl_2_·2H_2_O, 0.2; Na_2_MoO_4_·H_2_O, 0.002; FeCl_3_·6H_2_O, 0.01 and pH adjusted to 5.5 at 30°C and 200 rpm (Cavalcante and Dobereiner, 1988). Cells of *R. solanacearum* strains GMI1000, A21, and GMI1000-GFP were grown in blue-green (BG) medium (g L^−1^): casein peptone, 10.0; yeast extract, 1.0; casamino acids 1.0 at 28°C and 200 rpm (Boucher et al., 1985). Tetracycline (10 µg ml^−1^) was added to the medium for cultivation of the tetracycline-resistant *R. pseudosolanacearum* strain, GMI1000-GFP.

The bacterial strains and culture media used in this work are indicated in the **Supplementary Table 1**.

### Molecular Typing of Isolated *Ralstonia solanacearum* Strains From Argentina

Phylotype affiliation of the *R. solanacearum* strains was performed by multiplex PCR on the internal transcribed spacer region as described by Fegan and Prior (2005). Identification of phylotypes I, II, III, and IV was accomplished with four forward primers: Nmult 21:1F, Nmult 21:2F, Nmult23:AF, and Nmult 22: InF, respectively, and a common reverse primer Nmult 22:RR. Amplified fragments for each phylotype had an expected specific length (I: 144 bp, II:372 bp, III: 91 bp, and IV: 213 bp). The multiplex PCR also included a pair of primers common to all phylotypes (759/760). Amplifications were carried out in a total volume of 25-µl containing 1X PCR buffer, 0.2 mM of each deoxynucleoside triphosphate (dNTP), 1.5 mMMgCl_2_, 6 pmol of each phylotype-specific primer, 4 pmol of species-specific primers 759/760, 2 U of Taq DNA polymerase (Promega), and 50 ng of DNA template. Amplifications were performed in an automated Corbett thermocycler with an initial denaturation step at 96°C for 5 min; followed by 30 cycles of denaturation at 94°C for 15 s, annealing at 59°C for 30 s, and extension at 72°C for 30 s; with a final extension step at 72°C for 10 min. PCR products were analyzed by electrophoresis through 2% agarose gels and revealed under UV light.

The phylogenetic assignment of *R. solanacearum* strains was also determined based on analysis of the partial nucleotide sequences of the endoglucanase (*egl*) gene. PCR amplification of a 750-bp region of the *egl* gene was performed using the Endo-F and Endo-R primers pair as previously described (Fegan et al., 1998). Reactions were performed in a total in a total volume of 25 µl containing 1× DNA polymerase buffer, 1.5 mM MgCl2, 0.2 mM of each dNTP, 10 pmol of each primer, 1 U of Taq DNA polymerase (Promega), and 50 ng of DNA template. Amplification cycling conditions included an initial denaturation step at 96°C for 9 min; followed by 30 cycles of denaturation at 95°C for 1 min, annealing at 70°C for 1 min, and extension at 72°C for 2 min; with a final extension step at 72°C for 10 min. PCR products were purified and sequenced by Macrogen Services (Kumchun-ku, Seoul, Korea) using Endo-F and Endo-R primers. Forward and reverse chromatograms were edited using the Geneious v.7 software package. The determination of sequevars was assumed by *egl* sequence divergence values less than or equal to 1% (Fegan and Prior, 2005). Approximately maximum-likelihood phylogenetic trees were built including also sequences from worldwide reference strains representing the whole diversity of the *R. solanacearum* species complex. Phylogenetic and molecular evolutionary analyses were conducted in MEGA version X (Kumar et al., 2018).

### Plant Material, Growth Conditions, and Inoculation With *Gluconacetobacter diazotrophicus*

Seeds of wild-type *A. thaliana* Columbia-0 (Col-0) and *A. thaliana sid2* mutants were germinated on small plastic pots containing 33 g of soil: pearlite (90:10). Cultivation of plants occurred in a growth chamber at 22/24°C, 60% relative humidity with a photoperiod of 16 h light/8 h darkness for 14 days. For plant inoculation, *G. diazotrophicus* Pal5 cells were grown overnight (16 h) at 28°C in constant agitation at 200 rpm in liquid LGI-P medium. Cells were centrifuged at 12,000 ×g and the supernatant was discarded. Cells were washed twice in ultrapure sterile water and suspended in sterile distilled water to a final concentration of 10^6^ CFU/g of soil. Plants were inoculated by soil drenching with the adjusted suspension of *G. diazotrophicus* Pal 5 which was poured onto the rooted soil of a plant. Sterile water was used as negative control (Morel et al., 2017). Three independent growth chamber assays were performed using fourteen plant replicates of each treatment arranged in a complete randomized design. A total of 42 plants of each treatment were used to compare growth parameters. The pigment determination and counting colony-forming unit (CFU) assays were realized on three to six seedling replicates randomly selected of the three independent experiments.

### Colonization of *Arabidopsis thaliana* by *Gluconacetobacter diazotrophicus*

Colonization analyses were conducted at 28-day post-inoculation (dpi), using defined portions of roots and shoot soft six seedling replicates randomly selected of the three independent experiments. Each plant was carefully removed from the plastic cup and washed with sterile distilled water to take away the traces of soil. For tissue surface sterilization, the root and stem of each plant were immersed 70% v/v ethanol for 3 min under a laminar flow chamber. Then they were rinsed three times with sterile water and placed on a sterile absorbent paper. Extract of each organ was made under sterile conditions, cutting from each plant, 2 and 6 cm of stem and root, respectively, and were placed in a sterile Eppendorf tube containing 500 µl of NaCl 0.9% w/v. Then the tissue was mortared to obtain the corresponding extract. Twenty microliters of the extract and serial dilutions were plated in petri dishes with LGI-P agar 1.8% w/v supplemented with bromothymol blue (Cavalcante and Dobereiner, 1988; Reis et al., 1994; Baldani et al., 2014; Ferreira et al., 2017). The same samples were plated in petri dishes with LGI-P agar 1.8% w/v supplemented with antibiotics (chloramphenicol 20.0 mg/L, cycloheximide 150.0 mg/L). The plates were placed in an oven at 30°C for 7 days. After this time, colony forming units per g of plant tissues was determined (CFU/g).

To evaluate the effect of the colonization of the plants, the following growth parameters were measured: fresh weight of the different organs; length of the main root; length of the stem; number of rosette leaves; number of stem leaves; size of rosette leaves; size of stem basal leaves; size of leaves along the stem (Poupin et al., 2013; Poupin et al., 2016).

### Biocontrol *In Vivo* Assays of *Gluconacetobacter diazotrophicus* Against *Ralstonia pseudosolanacearum* GMI1000 and *Ralstonia solanacearum* A21

#### Inoculation of *Arabidopsis thaliana* Plants Containing *Gluconacetobacter diazotrophicus* With Phytopathogenic Strains of *Ralstonia solanacearum*

Inoculation with the phytopathogenic strains of the *R. solanacearum* species complex (GMI1000 or A21) was carried out in *A. thaliana* Col 0 and *sid2* mutant plants previously inoculated with the endophytic bacteria and in mock-inoculated plants. The inoculation was done by soil drenching, adding 1 mL of the bacterial suspension onto the rooted soil of plants. Previously, the roots were damaged with a tip to promote bacterial invasion of roots.

For plant inoculation, cells of the corresponding strains of *R. solanacearum* were grown overnight (16 h) at 28°C and under constant agitation at 200 rpm, in liquid BG medium. Bacterial cultures were diluted with sterile distilled water until a final concentration of 10^6^ CFU/g of soil was obtained. Gentamicin (10 µg ml^−1^) was added to the medium for cultivation of the gentamicin-resistant *R. pseudosolanacearum* strain, GMI1000-GFP.

A total of 144 plants in three independent experiments were used with a completely randomized design with six replicates per treatment. Three different phytopathogen strains were used. The following four treatments were applied: mock inoculated plants (*Gd* ^−^ *Rso* strain^−^): plants without bacteria; endophyte inoculated plants (*Gd* ^+^ *Rso* strain^−^): plants inoculated only with *G. diazotrophicus*; pathogenic bacterium inoculated plants (*Gd* ^−^ *Rso* strain^+^): plants inoculated only with *R. solanacearum*; endophyte and pathogenic bacterium inoculated plants (*Gd* ^+^ *Rso* strain^+^): plants inoculated with both bacteria. *Rso* strain: GMI1000/GMI1000-GFP/A21.

#### Colony-Forming Unit Counting Assays

Determination of microbial population in plant organs was performed at 28 days post-inoculation with *G. diazotrophicus* and 12 dpi with the strains GMI1000 and A21 of *R. solanacearum*, using defined portions of roots and shoots. For tissue surface sterilization of plants and determination of *G. diazotrophicus* population, the procedure described in *Colonization of Arabidopsis thaliana by Gluconacetobacter diazotrophicus* was followed.

Determination of CFU of different strains of *R. solanacearum* was carried out by plating 20 µl of the extract and serial dilutions in petri dishes with modified sorbitol MacConkey agar (mSMSA) agar 1.8% w/v supplemented with 0.05 g/L of 2,3,5-triphenyltetrazolium chloride, TTC (French et al., 1995) containing the following (g L^−1^): casamino acids, 1.0; peptone 10.0; glucose 5.0. The following antibiotics were added to medium:5 mg/L chloramphenicol, 100 mg/L cycloheximide, 0.5 mg/L penicillin-G, 5 mg/L crystal violet, and 2,3,5-triphenyl tetrazolium chloride. The plates were placed in an oven at 28°C for 7 days. After this time, CFU/g was determined. Six seedling replicates of each treatment of three independent experiments were used (Engelbrecht, 1994 modificate by Elphinstone et al., 1996).

### Anatomical Studies to Determine Structural and/or Physiological Changes

#### Inclusion in Paraffin and Differential Staining

Different treatments samples from tissue *A. thaliana* Col 0 and *sid2* mutant plants were taken at 14, 20, and 28 dpi with *G. diazotrophicus* and 12 dpi with *R. solanacearum* strains and were fixed in FAA solution (50% ethanol, 5% glacial acetic acid, 30% formaldehyde, 15% water). Different organs of each treatment were separated and dehydrated with ethanol and ethanol/xylene of ascending concentration solutions. Then they were embedded in paraffin and roots, stems, and leaf blades were cut at 10 µm thickness with Minot microtome and stained with safranin-fast green. Samples were mounted in Canada balsam natural (Biopack). For polychromatic dye with toluidine blue solution (0.05% w/v), cuts manually obtained were incubated in the dye during 5 min, washed with distilled water, and mounted with glycerol-water (50% v/v) (D´Ambrogio, 1986). Observations were made with a light microscope (Zeiss MC 80 Axiolab) equipped with a camera.

#### Confocal Microscopy

Cross and longitudinal sections of fresh root and stem samples (from plants of four treatments) obtained with the technique of freehand cutting were mounted on a slide, surrounded with solid Vaseline and covered with agarose (1% w/v) used as a mean of immersion and fixation. Samples were observed using a confocal microscope (Nikon C1SiR attached on a Nikon TE2000 inverted microscope). Three independent experiments were performed.

#### Callose Detection

The detection of callose deposits was carried out in *A. thaliana* Col 0 and *sid2* mutant plants of the four treatments described above with *R. solanacearum* GMI1000 strain. Three replicates of plants were randomly selected. Three independent experiments were performed. Roots and leaves of these plants were kept overnight in alcohol 96°; once the organs were completely decolorated, they were incubated in sodium phosphate buffer (0.07 M, pH 9) for 30 min and then in an aniline blue solution (0.05% w/v) for 60 min (Daurelio et al., 2009). Finally, the samples were mounted in a glycerol-water mixture (50% v/v) and observed immediately using an Epi-fluorescence-UV microscope (MIKOBA F320 with mercury lamp power box).

### Pigment Determination

To evaluate potential changes of pigments in *A. thaliana* plants, the content of chlorophyll *a* (Chl a), chlorophyll *b* (Chl b) and total chlorophyll (Chl a + b) was determined at 28 dpi with *G. diazotrophicus* and 12 dpi with *R. pseudosolanacearum* GMI1000 or *R. solanacearum* A21. *A. thaliana* seedlings were treated with *G. diazotrophicus* and/or *R. pseudosolanacearum* GMI1000, *G. diazotrophicus*, and/or *R. solanacearum* A21 12 dpi and mock inoculated plants. For this quantification, two leaves per plant of three plant replicates from each treatment were used. The experiments were performed independently by triplicate. For the quantification of chlorophylls, two leaf discs of 0.8 cm diameter were cut and incubated in 1 ml of N,N-dimethylformamide (DMF) for 72 hs at room temperature and in the dark. The absorbance of the samples was then measured at 664 and 647 nm. The content of total chlorophyll, chlorophyll a, and chlorophyll b were determined according to the equations described by Porra (2002).

### Data Analysis

For comparison between two treatments, Shapiro-Wilk normality test was performed, and Student’s t or Mann Whitney test was applied. For comparison of a larger number of treatments an analysis of variance (ANOVA) and multiple comparisons test were performed. It was considered statistically significant for *p* < 0.05. The data obtained were processed with the InfoStat program (2014). Chloroplast length values were obtained from 10 fields/leaf cross sections; using 5 different sections. Chloroplast length values were obtained using the ImageJ processing program.

## Results

### *Gluconacetobacter diazotrophicus* Induces Anatomical Changes in *Arabidopsis thaliana* Seedlings

Endophytic bacterial population in roots and stems was analyzed at 28 dpi to corroborate the presence of *G. diazotrophicus* in *A. thaliana* seedlings. CFU counting assays revealed that the endophytic population within the root and stem of *A. thaliana* was (2.57 ± 0.21) x10^5^ CFU/g and (2.60 ± 3.66) x10^5^ CFU/g, respectively. The colonization of endophytic bacteria was tested in roots of *A. thaliana* Col 0 using a magnifying glass. The observation of inoculated seedlings showed some colonies associated with the root, mainly in the places where the lateral roots emerge; in the vicinity of the radical apex and in the radical hairs (**Supplementary Figures 4C, D**). The roots of the mock inoculated plants did not show associated bacterial colonies (**Supplementary Figures 4 A, B**). Radical hairs with associated colonies showed no visible damage (**Supplementary Figure 4D**).

**Figure 1:**
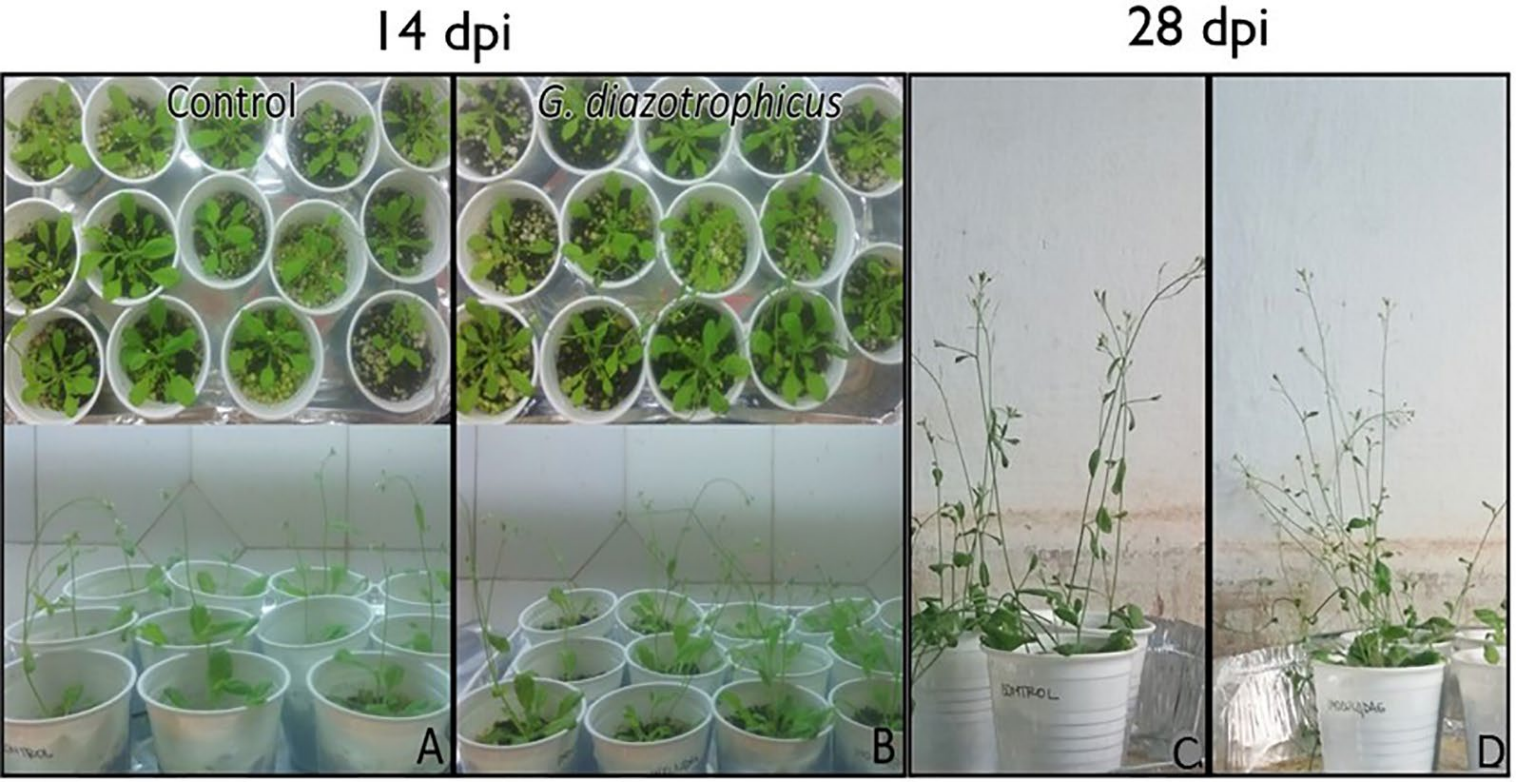
*Arabidopsis thaliana* plants inoculated with *Gluconacetobacter diazotrophicus* and mock inoculated plants at 14 and 28 dpi. **(A**–**C)** Mock inoculated plants. **(B**–**D)** Inoculated plants. Three independent growth chamber assays were performed using 14 plant replicates of each treatment.

**Figure 2:**
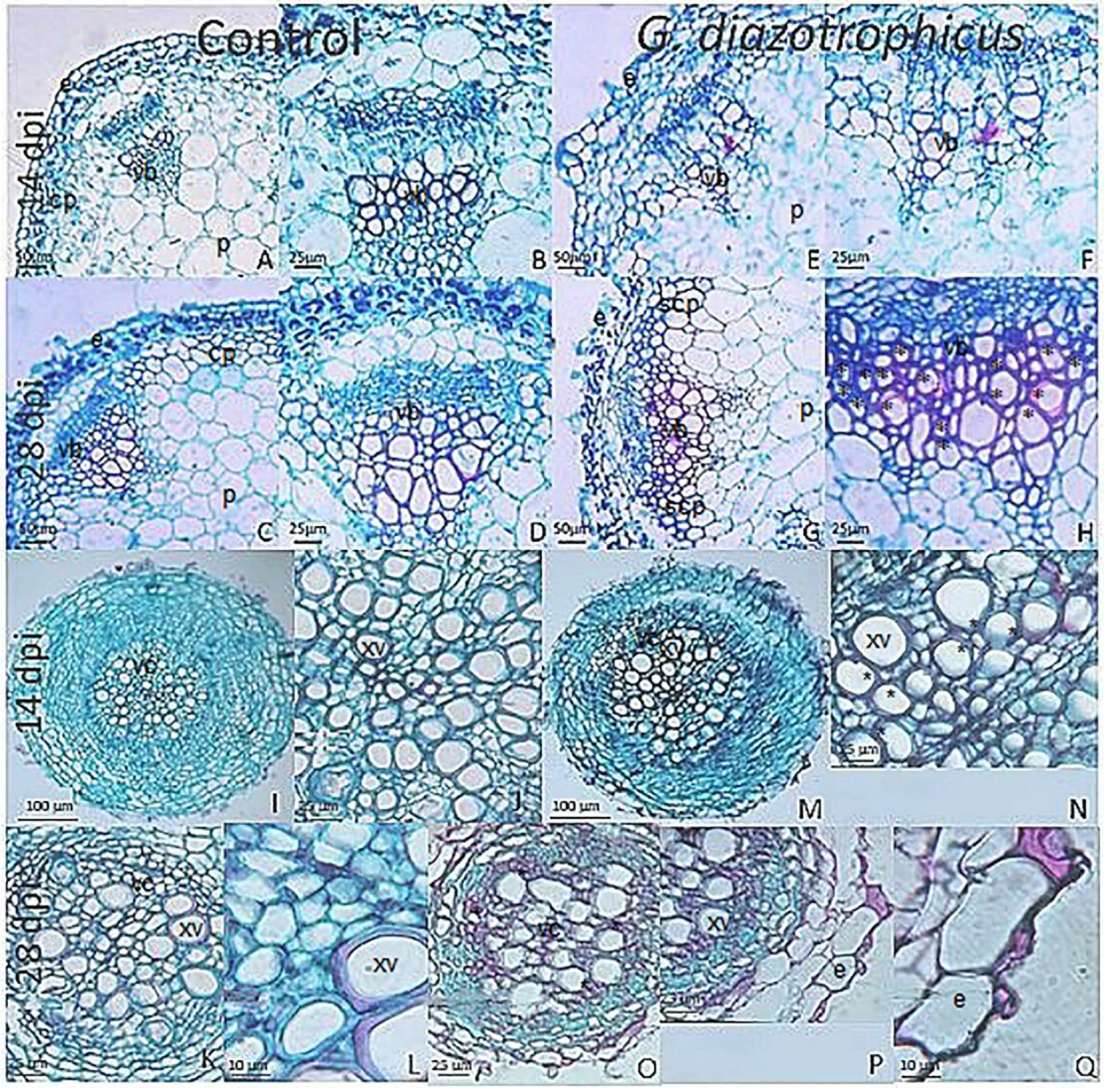
Micrographs of cross sections of Arabidopsis thaliana plants inoculated with Gluconacetobacter diazotrophicus at different dpi stained with safranin-fast green. **(A**–**H)** Stem; **(I**–**Q)** Root. **(A**–**D**, **I**–**L)** Mock inoculated plants; **(E**–**H**, **M**–**Q)** inoculated plants. **(B**–**D)**, Stem vascular bundle detail of 14 and 28 dpi mock inoculated plants **(A**–**C)** showing less lignification and amount of xylematic tissue than in inoculated plants at 28 dpi. **(F**–**H)**, Stem vascular bundle detail of 14 and 28 dpi inoculated plants **(E**–**G)**. **(H)** shows a greater lignification and amount of xylematic tissue than in mock inoculated plants. **(J)**, Root vascular cylinder detail of mock inoculated plants 14 dpi **(I)** showing smaller diameter and lignification of the xylematic vessels. **(N)**, Root vascular cylinder detail of inoculated plants 14 dpi **(M)** showing grater diameter and lignification of the xylematic vessels. **(L)**, Root vascular cylinder detail of mock inoculated plants 28 dpi **(K)** showing low lignification of the xylematic vessels. **(O)**, Root vascular cylinder of inoculated plants 28 dpi showing greater lignification of the xylematic vessels. **(Q)**, Root epidermis detail of inoculated plants 28 dpi **(P)** showing sclerosis of tissue. cp, cortical parenchyma; e, epidermis; p, pith; scp, sclerosed cortical parenchyma; vb, vascular bundle; vc, vascular cylinder; xv, xylematic vessels. Asterisk indicated more xylematic vessels lignification. Each image is a representative result of observation of at least 10 section from five biological replicates.

**Figure 3:**
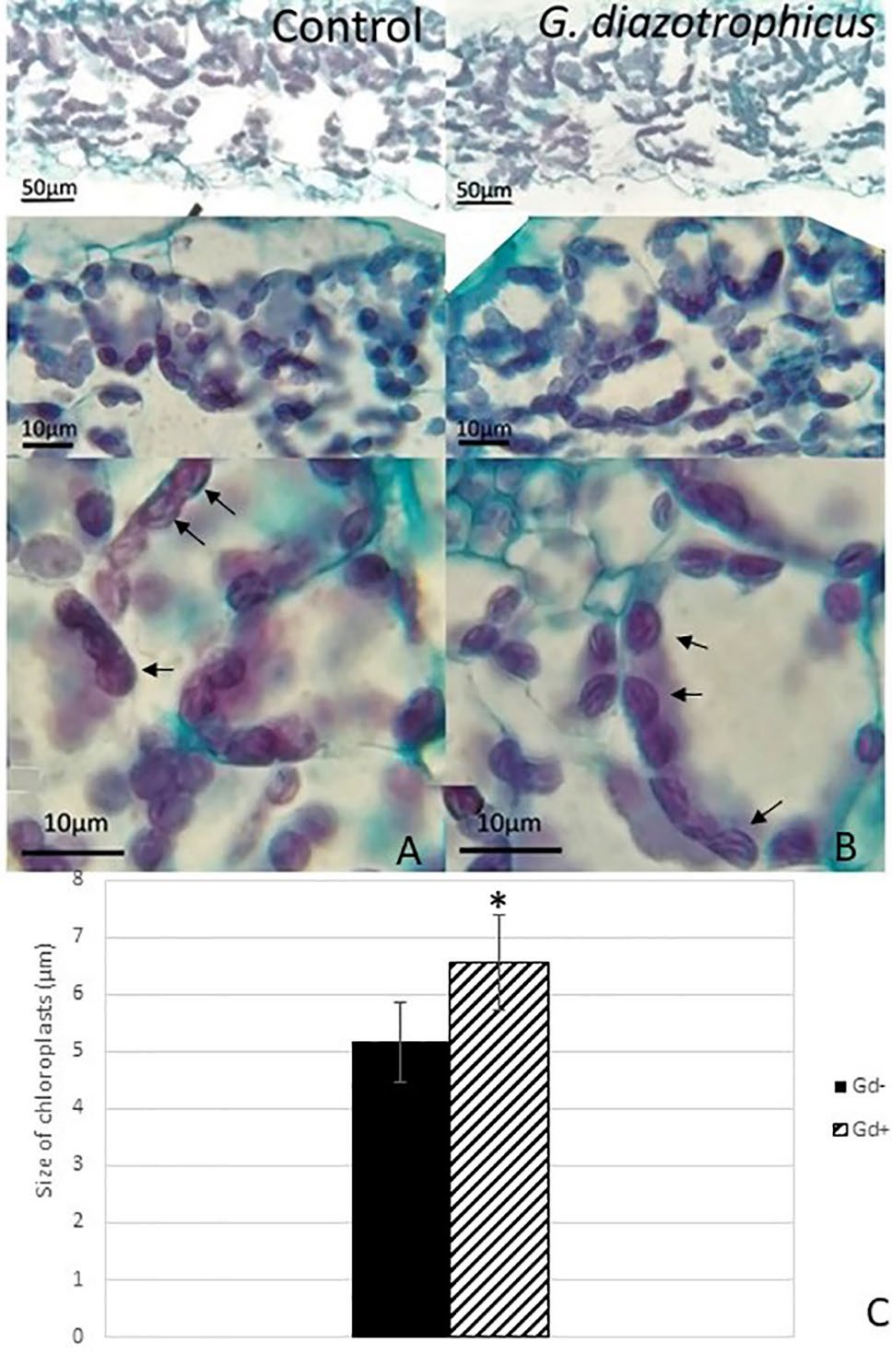
Micrograph of cross sections of *Arabidopsis thaliana* leaf inoculated with *Gluconacetobacter diazotrophicus* at 28 dpi. Images are displayed at different magnifications. **(A)** Mock inoculated plants; **(B)** inoculated plants; the black arrows indicate chloroplasts; **(C)** bar graph comparing the size of chloroplasts between inoculated plants and mock inoculated plants; chloroplast size values were obtained from 10 fields/leaf cross sections; using five different sections. The error bars represent the standard deviation. Significant differences between treatments are indicated by an asterisk (Student’s t test, *p* < 0.05).

**Figure 4:**
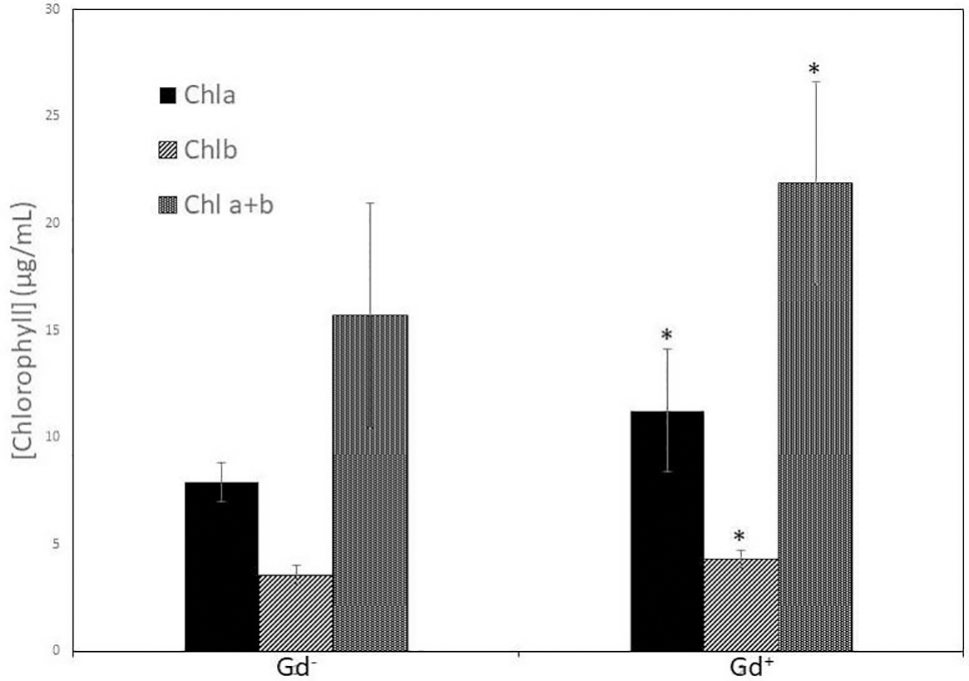
Bar graph showing the concentration of chlorophyll *a*, chlorophyll *b*, and chlorophyll *a + b* in leaves of *Arabidopsis thaliana* inoculated with *Gluconacetobacter diazotrophicus* at 28 dpi. Concentration of pigments values are means of two leaves per plant of three plant replicates from each treatment. The experiments were performed independently by triplicate. The error bars represent the standard deviation. Significant differences between treatments are indicated by an asterisk (Student’s t test, *p* < 0.05).

Despite of not having exomorphological changes between 28 dpi inoculated plants and mock inoculated controls (**Figure 1** and **Supplementary Figure 1**), endomorphological analysis showed that *G. diazotrophicus* colonizes and promotes significant anatomical modifications in *A. thaliana* Col 0 plants inoculated with 10^6^ CFU/g of soil. These were observed at 28 dpi in root (**Figures 2O–Q**) and stem (**Figures 2G, H**) and only in root at 14 dpi (**Figures 2M, N**). Roots inoculated with *G. diazotrophicus* 14 and 28 dpi, presented an increase in the diameter and a greater lignification of the xylem vessels. Epidermis, exodermis, endodermis, and pericycle of the main root sclerosis was also part of the observed structural features in plants inoculated with *G. diazotrophicus* after 28 days (**Figures 2P, Q**). Stems (**Figures 2G, H**) showed an increase in xylem tissue and in the amount of sclerosed cortical parenchyma tissue between the vascular bundles with respect to the mock inoculated controls (**Figures 2C, D**). Greater lignification of the xylem was also observed in inoculated plants. There were not significant structural changes between the leaves of inoculated 28 dpi and control plants (**Figures 3A, B**). Nevertheless, changes were detected in the chloroplasts between treatments. Although there was no increase in quantity, a greater size of chloroplasts was observed in the inoculated plants 28 dpi (6.56 ± 0.83 µm) with respect to the mock inoculated controls (5.16 ± 0.71 µm) (**Figure 3C**). Values of chlorophyll *a*, chlorophyll *b*, and chlorophyll *a + b* in plants inoculated with *G. diazotrophicus* at 28 dpi revealed significantly higher amount of pigments in plants inoculated with the endophytic bacteria than the control plants (**Figure 4**). We also tested the lignin presence in *A. thaliana sid2* mutants plants and the presence of lignin thickening caused by inoculation of endophytic bacteria was not observed in any tissue (stems and root) of these mutant plants (**Supplementary Figure 7**). *A. thaliana sid2* mutant accumulate much less SA in comparison to wild-type plants since this plant mutant is deficient in the induction of SA accumulation having blockage the SA biosynthesis.

### *Gluconacetobacter diazotrophicus* Activates Plant Defense Protecting *Arabidopsis thaliana* Seedlings From the Invasion of *Ralstonia pseudosolanacearum* GMI1000

To analyze the possible antagonistic effects of the *G. diazotrophicus* Pal5 strain in the presence of the *R. pseudosolanacearum* GMI1000 strain, experiments were performed on *A. thaliana* plants during biotic stress produced by the phytopathogenic bacterium.

**Figure 5:**
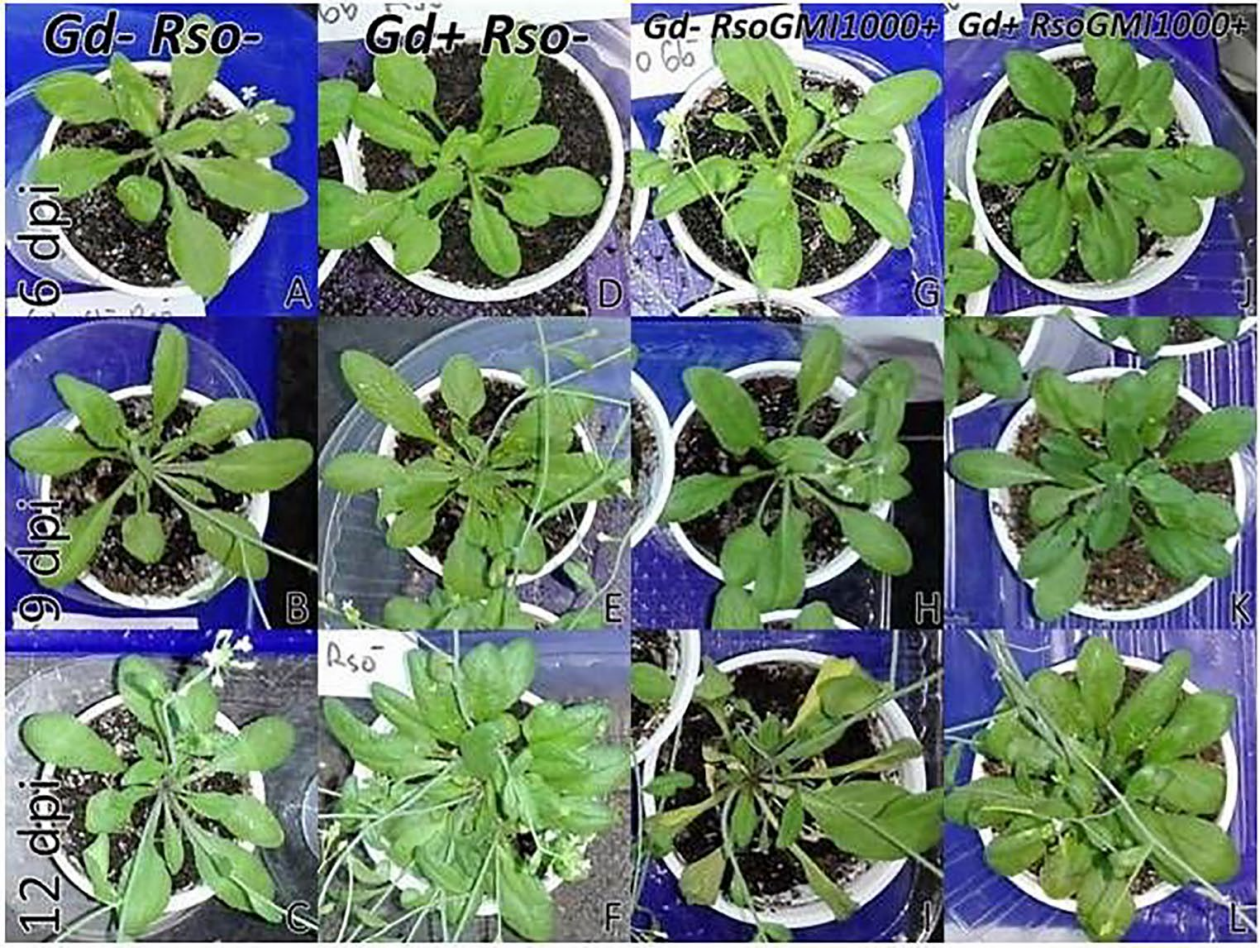
Plants of *Arabidopsis thaliana* grown with different treatments, images are shown at different post-inoculation days (6, 9, and 12) with *Ralstonia pseudosolanacearum* GMI1000. Three independent growth chamber assays were performed using six replicate plants of each treatment. **(A**–**C)** Mock inoculated plants; **(D**–**F)** plants inoculated with *G. diazotrophicus* Pal5; **(G**–**I)** plants inoculated with *R. pseudosolanacearum* GMI1000; **(J**–**L)** plants inoculated with *G. diazotrophicus* and with *R. pseudosolanacearum* GMI1000.

At 6 and 9 dpi, *A. thaliana* seedlings inoculated only with *R. pseudosolanacearum* GMI1000 (**Figures 5 G, H**) did not show exomorphological differences regarding to those plants inoculated with *G. diazotrophicus* and *R. pseudosolanacearum* GMI1000 (**Figures 5J, K**), inoculated only with *G. diazotrophicus* (**Figures 5D, E**), or mock inoculated controls (**Figures 5A, B**). At 12 dpi, exomorphological changes arose in those plants only inoculated with *R. pseudosolanacearum* GMI1000. The leaves of the rosette were chlorotic and dehydrated (**Figure 5I**).

CFU counting assays were performed to evaluate bacterial number (CFU/g) of *G. diazotrophicus* (28 dpi) and *R. pseudosolanacearum* GMI1000 (12 dpi) of respective *A. thaliana* plants treatments. The results are indicated in **Table 1**. In roots of *A. thaliana* plants *R. pseudosolanacearum* GMI1000 population decreased from (9.40 ± 0.50) x10^10^ CFU/g in the absence of *G. diazotrophicus* to (8.23 ± 1.90) x10^6^ CFU/g, when is confronted to the endophyte. Stem extracts and dilutions of *A. thaliana* plants with *G. diazotrophicus* did not show *R. pseudosolanacearum* GMI1000 growth in the selective medium mSMSA (Elphinstone et al., 1996).

**Table 1:** Counting values of *Gluconacetobacter diazotrophicus* and *Ralstonia solanacearum* in roots and stems of *Arabidopsis thaliana* plants.

	*CFU counting per gram of organ*			
	*Root*		Stem	
	*LGI-P medium for Gd counting*	*mSMSA medium for Rso counting*	*LGI-P medium for Gd counting*	*mSMSA medium for Rso counting*
*Gd-Rso-*	n.d.	n.d.	n.d.	n.d.
*Gd+Rso-*	(2.57 ± 0.21) x10^5^a	n.d.	(2.60 ± 3.66) x10^5^a	n.d.
*Gd-RsoGMI1000+*	n.d.	(9.40 ± 0.50) x10^10^b	n.d.	(3.20 ± 2.10) x10^9^
*Gd-RsoA21+*	n.d.	(1.40 ± 0.68) x10^7^b´	n.d.	(3.79 ± 4.82) x10^5^
*Gd+ RsoGMI1000+*	(5.91 ± 3.16) x10^5^a	(8.23 ± 1.90) x10^6^c	(6.10 ± 2.70) x10^4^a	n.d.
*Gd+ RsoA21+*	(5.17 ± 6.48) x10^5^a	(1.92 ± 2.31) x10^5^c´	(5.50 ± 3.20) x10^4^a	n.d.

n.d., not detected.

Data expressed as mean averages of six seedling replicates of three independent experiments ± standard error. Shapiro-Wilk normality test was performed, and Student’s t or Mann Whitney test was applied. Different letters indicate significant differences in counts (p<0.05).

Although exomorphological alterations were not observed in inoculated seedlings at 6 and 9 dpi, stem longitudinal sections stained with toluidine blue (1% w/v) showed the presence of *R. pseudosolanacearum* GMI1000 along the stem, but only in those plants that were not previously inoculated with *G. diazotrophicus* (**Supplementary Figures 2A–H**). In plants previously inoculated with *G. diazotrophicus*, the presence of phytopathogenic bacteria was not observed (**Supplementary Figures 2I–L**).

**Figure 6:**
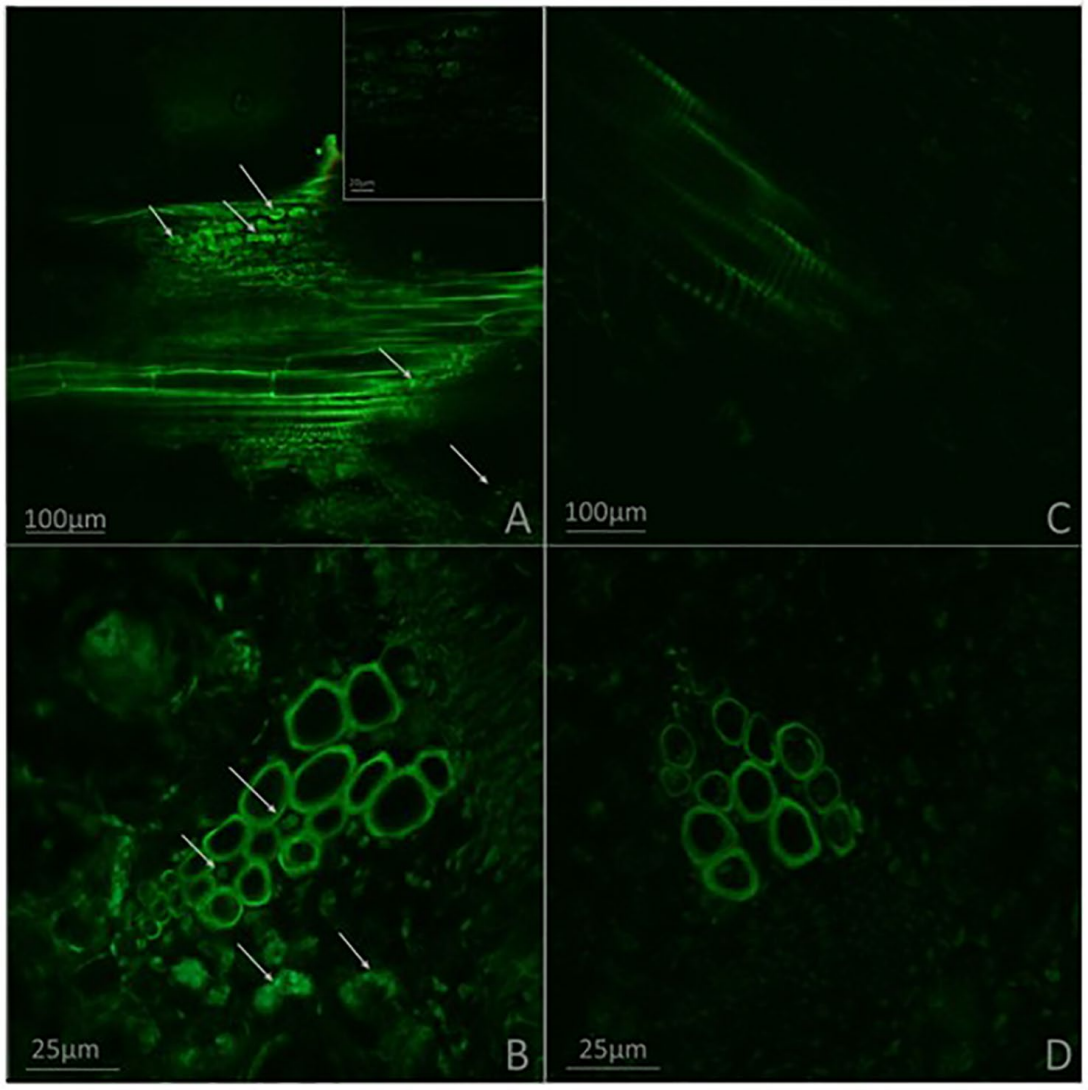
Micrographs obtained by confocal microscopy. **(A**–**B)** Plants of Arabidopsis thaliana inoculated with Ralstonia pseudosolanacearum GMI1000-GFP 12 dpi. **(C**–**D)** Plants of A. thaliana inoculated with G. diazotrophicus 28 dpi and R. pseudosolanacearum GMI1000-GFP 12 dpi. **(A**, **C)** Stem longitudinal section. **(B**, **D)** Stem cross section. The white arrows indicate the presence of R. pseudosolanacearum. Each image is a representative result of observation of at least 10 section from three biological replicates.

**Figure 7:**
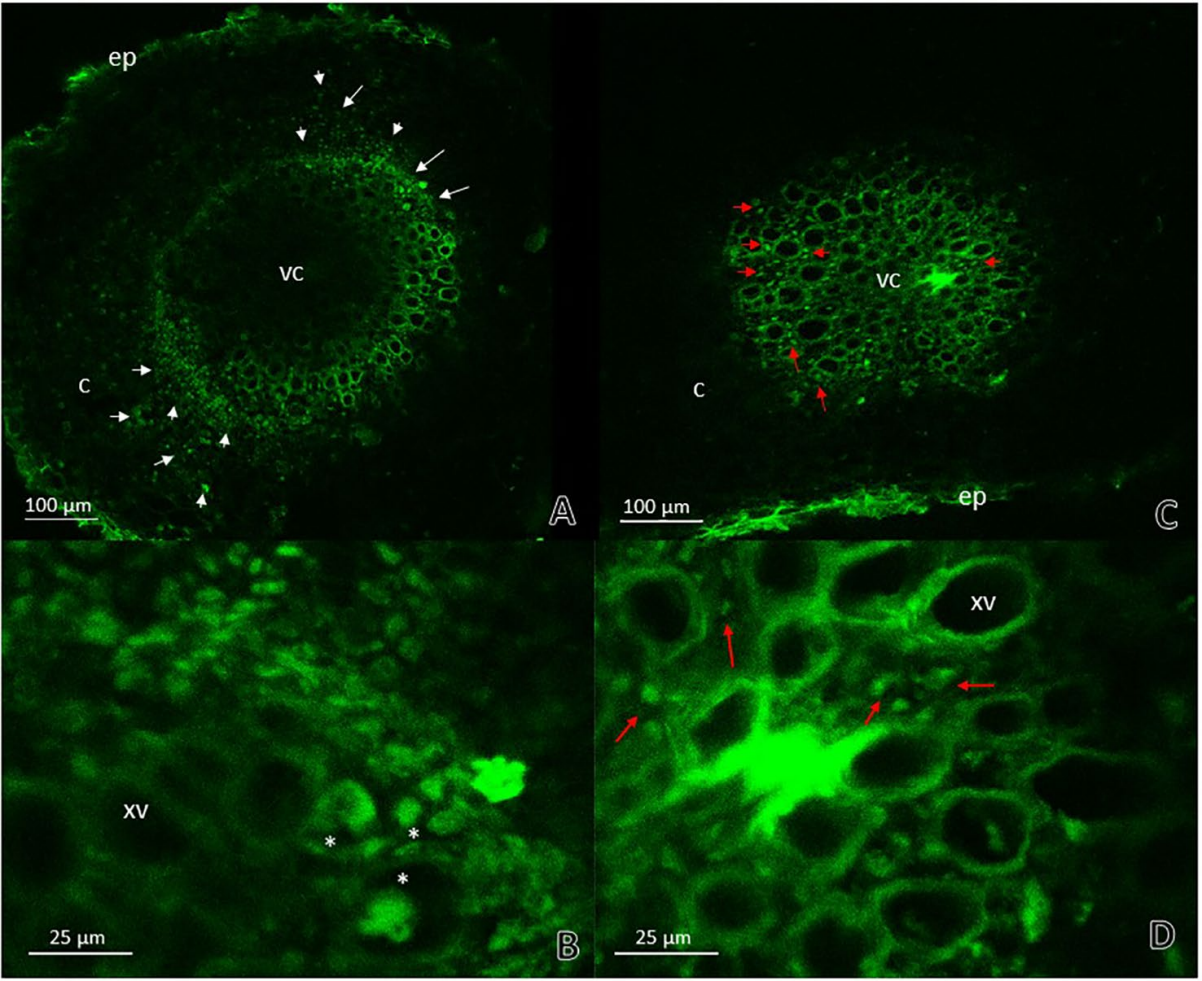
Root cross sections micrographs obtained by confocal microscopy. **(A)** Plants of Arabidopsis thaliana inoculated with Ralstonia pseudosolanacearum GMI1000-GFP 12 dpi. The white arrows indicate the pathogenic bacteria presence in areas of the cortical zone and endodermis next to vascular cylinder and in the xylematic vessels. **(B)** Root vascular cylinder detail of panel **(A)** showing xylematic vessels. White asterisks show xylematic vessels filled with pathogenic bacteria. **(C)** Plants of A. thaliana inoculated with G. diazotrophicus 28 dpi and R. pseudosolanacearum GMI1000-GFP 12 dpi. **(D)** Root vascular cylinder detail of panel **(C)** showing xylematic vessels. The red arrows indicate the pathogenic bacteria presence in areas surrounding xylematic vessels. c, cortical zone; ep, epidermis; xv, xylematic vessels; vc, vascular cylinder. Each image is a representative result of observation of at least 10 section from three biological replicates.

To corroborate the previous results and to confirm the presence or absence of the phytopathogenic bacterium, *A. thaliana* seedlings were inoculated with an *R. pseudosolanacearum* GMI1000-GFP strain. Stems and roots of plants previously inoculated with *G. diazotrophicus* and plants without *G. diazotrophicus* were cut in cross and longitudinal sections and confocal microscopy technique was applied. This experiment allowed locating the bacterium within the plant tissue. The results observed agree with those previously obtained. Phytopathogenic bacterium colonization in seedlings *Gd* ^−^ *RsoGMI1000-*GFP^+^ was observed in the xylem tracheary elements as well as the parenchyma cells in the cortex at 12 dpi (**Figures 6A, B**). The presence of *R. pseudosolanacearum* GMI1000-GFP was not manifest in plant stems previously inoculated with *G. diazotrophicus* (**Figures 6C, D**). These results show that bacterial colonization is restricted in stems of seedlings that previously were inoculated with *G. diazotrophicus*. *R. pseudosolanacearum* GMI1000-GFP was observed in root cross section in both treatments showing correspondence with CFU counting assays (**Figure 7**, **Table 1**). Strikingly, in those plants without *G. diazotrophicus* (*Gd* ^−^ *RsoGMI1000-GFP* ^+^), bacteria were localized with a broader colonization in cortical zone proximal to the vascular cylinder and several of xylem vessels were filled (**Figures 7A, B**). Contrary to this, in seedlings *Gd* ^+^ *RsoGMI1000-GFP* ^+^, the *R. pseudosolanacearum* GMI1000-GFP distribution seems to be different. Bacteria seem to be surrounding the xylematic vessels probably in the xylem parenchyma cells. No large colonization of *R. pseudosolanacearum* GMI1000-GFP was observed in the cortical zone of the root. Thus, bacterial invasion of the vascular cylinder appears restricted and larger metaxylem elements are not significantly colonized (**Figures 7C, D**).

**Figure 8:**
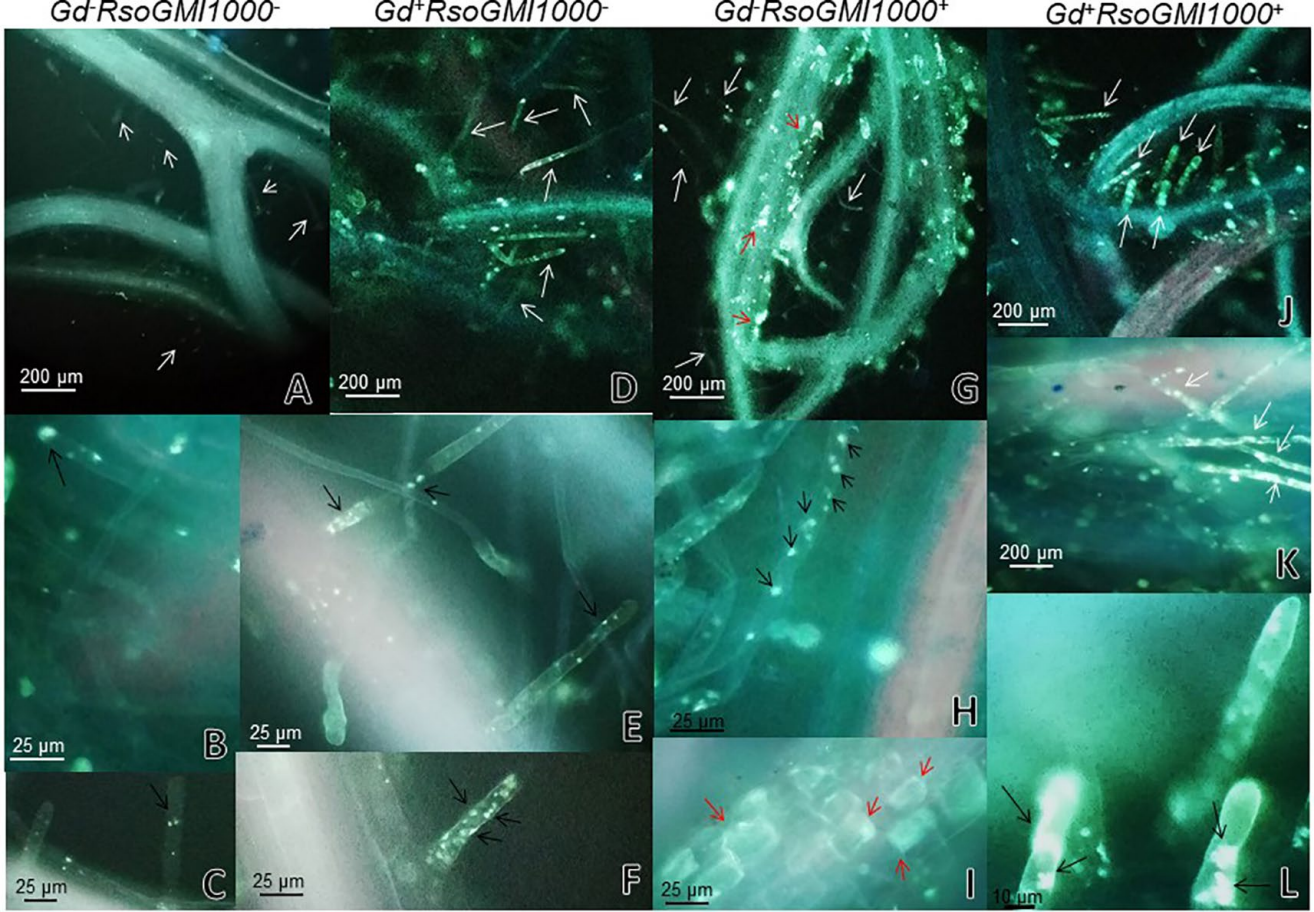
Observation of callose deposits with epifluorescence microscope in roots of Arabidopsis thaliana. In each column the treatment is specified. The white arrows indicate root hairs. The black arrows indicate root hair papillae. The red arrows indicate zones and cells of the root surface with callose. The experiments were performed at 12 dpi with pathogenic bacterium. **(A**–**C)** Mock inoculated plants; **(D**–**F)** plants inoculated with Gluconacetobacter diazotrophicus Pal5; **(G**–**I)** plants inoculated with Ralstonia pseudosolanacearum GMI1000; **(J**–**L)** plants inoculated with G. diazotrophicus and with R. pseudosolanacearum GMI1000. Each image is a representative result of observation of at least 10 section from three biological replicates.

**Figure 9:**
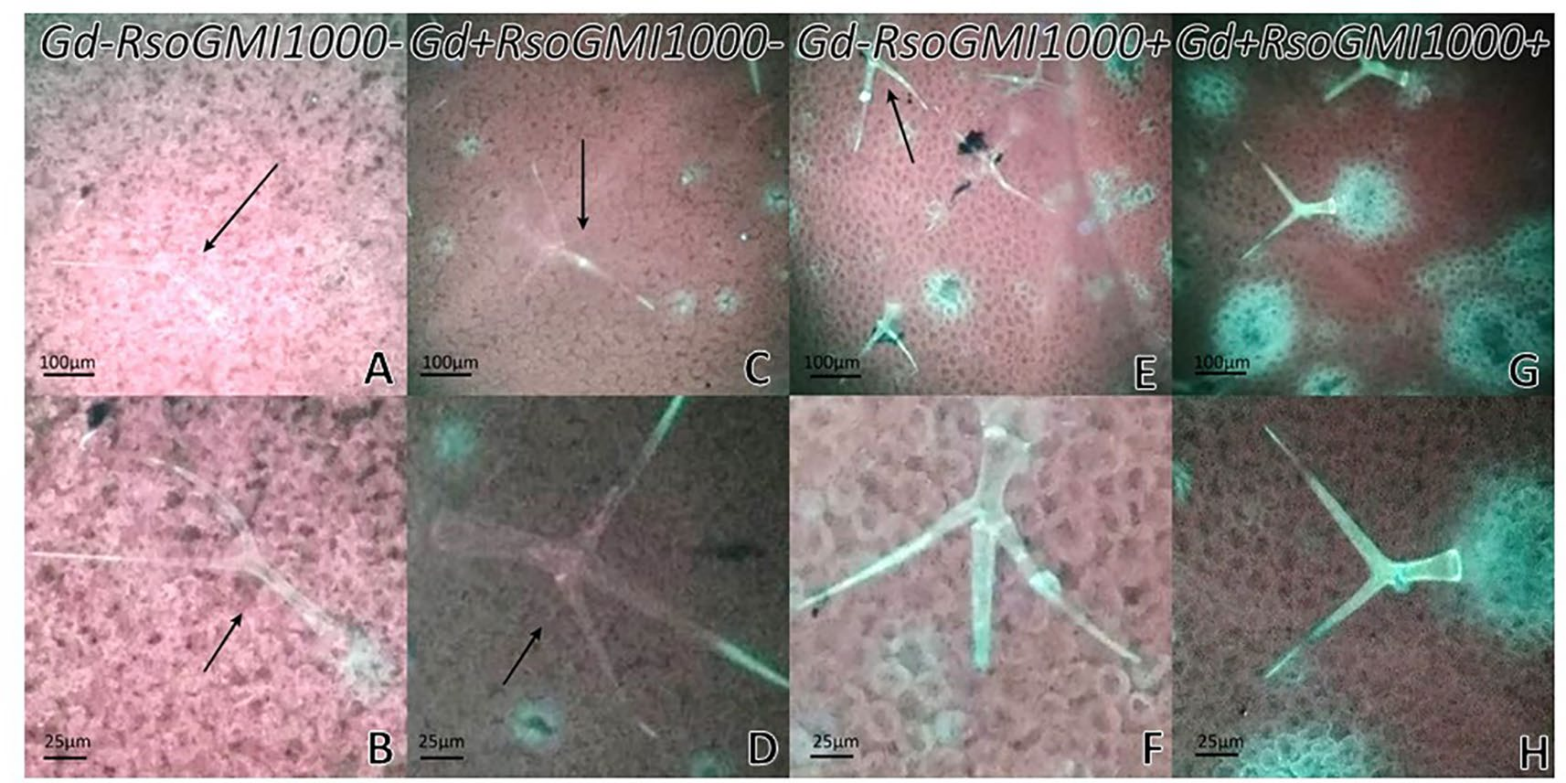
Observation of callose with epifluorescence microscope in leaves of Arabidopsis thaliana. In each column the treatment is specified. The black arrows indicate trichomes. The experiments were performed at 12 dpi with pathogenic bacterium. **(A**–**B)** Mock inoculated plants; **(C**–**D)** plants inoculated with Gluconacetobacter diazotrophicus Pal5; **(E**–**F)** plants inoculated with Ralstonia pseudosolanacearum GMI1000; **(G**–**H)** plants inoculated with G. diazotrophicus and with R. pseudosolanacearum GMI1000. Each image is a representative result of observation of at least 10 section from three biological replicates.

Effects of *G. diazotrophicus* on the cellular defense response mediated by callose deposition were investigated. Callose was localized using aniline blue solution leading to yellow fluorescence. Papillae are appositions that reinforce cell wall at sites of interaction with pathogenic microorganism. These papillae structures were observed at discrete sites of root hairs in plants inoculated with *G. diazotrophicus* or *R. Pseudosolanacearum* GMI1000, indicating that in both treatments callose deposition occurs (**Figures 8D–I**). The callose deposits were clearly increased in those plants infected with *R. pseudosolanacearum* GMI1000 which had previously been inoculated with *G. diazotrophicus* (**Figures 8J–L**). Similarly, newly callose deposits were accumulated within leaf tissues and trichomes in those seedlings previously inoculated with *G. diazotrophicus* and subsequently with *R. pseudosolanacearum GMI1000* (**Figure 9**). This assay was also performed using *A. thaliana sid2* mutants. No callose deposition was observed in plants inoculated with *G. diazotrophicus* or *R. Pseudosolanacearum* GMI1000 or both bacteria in trichomes or portions of the epidermis of these seedlings (**Supplementary Figures 5B–K**). No deposits of callose were observed in the papillae of the radical hairs of any of the treatments tested in the roots of the *A. thaliana sid2* mutant seedling (**Supplementary Figures 6B–K**).

**Figure 10:**
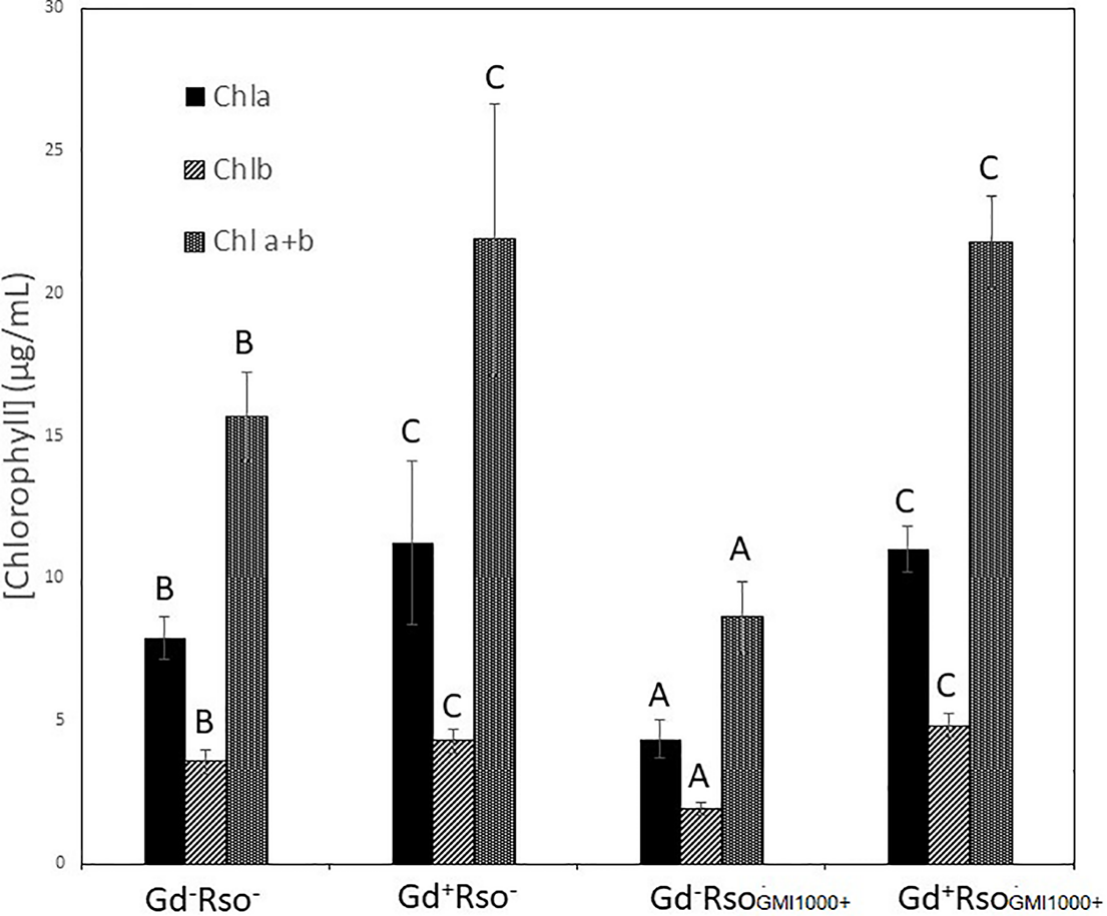
Bar graph showing the concentration of chlorophyll *a*, chlorophyll *b*, and chlorophyll *a + b* in leaves of *Arabidopsis thaliana* inoculated 28 dpi with *Gluconacetobacter diazotrophicus* (Gd^+^Rso^−^), inoculated 12 dpi with *Ralstonia pseudosolanacearum* GMI1000 (Gd^−^RsoGMI1000^+^), with both bacteria (Gd^+^ RsoGMI1000^+^) or mock inoculated plants (Gd^−^Rso^−^). Concentration of pigments values are means of two leaves per plant of three plant replicates from each treatment. The experiments were performed independently by triplicate. The error bars represent the standard deviation. Significant differences between treatments are represented by different letters (ANOVA, *p* < 0.05).

There were significant statistical differences in the amount of pigments between those plants only exposed to *R. pseudosolanacearum* GMI1000 and those pretreated with *G. diazotrophicus*. No variation in the plant pigments between *Gd* ^+^ *RsoGMI1000* *^+^* and *Gd* ^+^ *RsoGMI1000* *^−^* treatments was observed. Pigments concentrations were higher in plants treated with *Gd* ^+^ *RsoGMI1000* *^+^* and *Gd* ^+^ *RsoGMI1000* *^−^* than in control (*Gd* ^−^ *RsoGMI1000* ^−^) for both treatments. Chlorophyll *a*, chlorophyll *b*, and chlorophyll *a + b* concentrations decreased in those plants only inoculated with *R. pseudosolanacearum GMI1000* at 12 dpi (*Gd* ^−^ *RsoGMI1000* ^+^) (**Figure 10**).

### *Gluconacetobacter diazotrophicus* Successfully Controls the Infection of *Arabidopsis thaliana* Seedlings Inoculated With an Argentine Isolation of the Bacterium *Ralstonia solanacearum* A21

Three strains were isolated from infected tomato (A11, A21) and pepper (A31) plants from the Argentina north eastern region. These strains were characterized as *R. solanacearum* using microbiological and *in planta* tests. The genomic DNA from the three *R. solanacearum* strains was obtained and using for the genotypic identification. Phylotype-specific multiplex PCR analyses amplified the expected 280 and 372 bp fragments, indicating that all three isolates belonged to the *R. solanacearum* phylotype II. Based on phylogenetic analysis of partial *egl* sequences strains from Argentina were assigned to the phylotype IIA sequevar 50 (strains A11 and A21) and sequevar 38 (strain A31) (**Supplementary Figure 3**). Both sequevars are associated to strains isolated from solanaceous crops in South America. Since all the Argentinean strains presented a similar tomato symptom, we decided to work with the one of them, named *R. solanacearum* A21.

**Figure 11:**
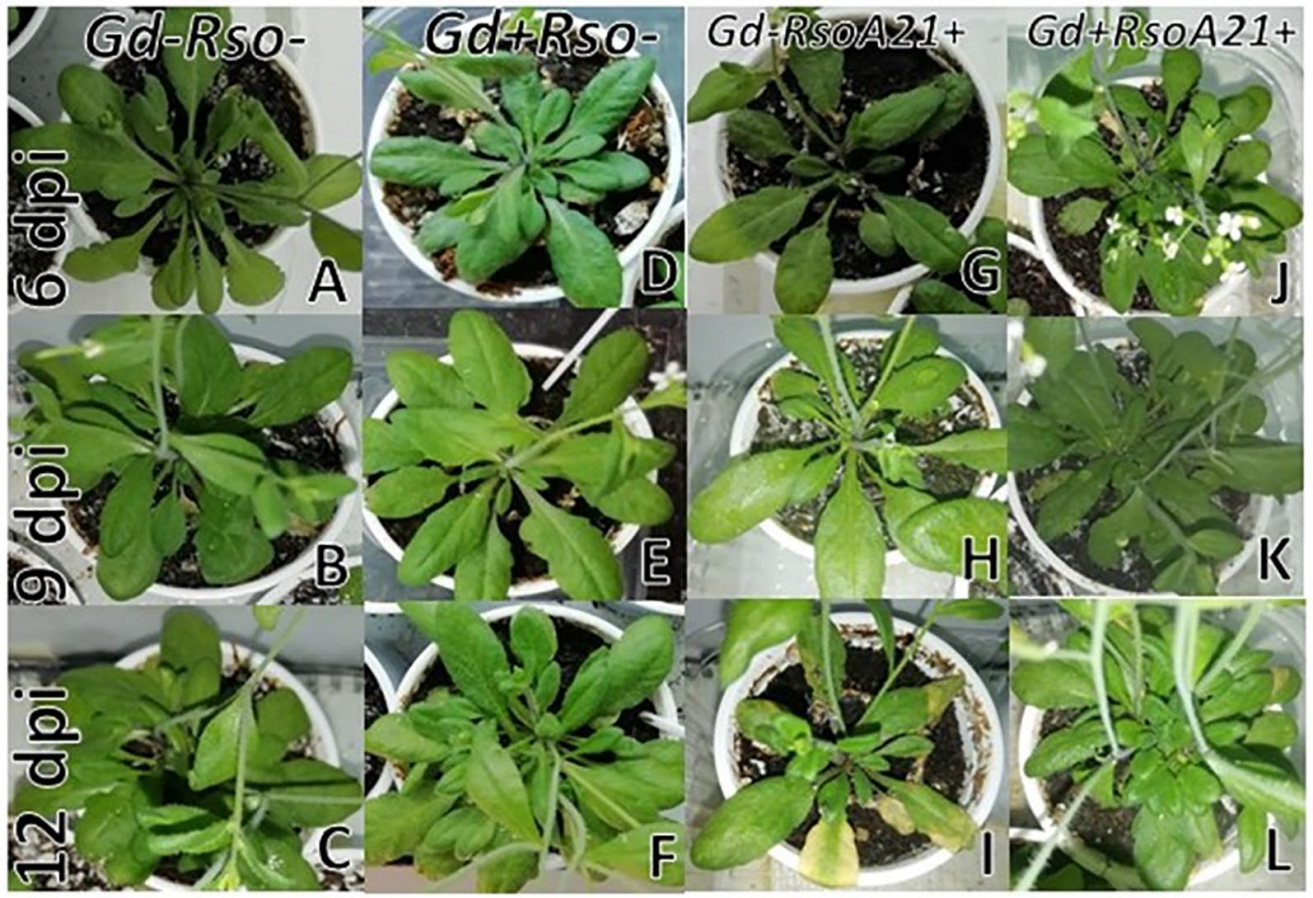
Plants of *Arabidopsis thaliana* grown with different treatments, images are shown at different post-inoculation days (6, 9, and 12) with *Ralstonia solanacearum* A21. Three independent growth chamber assays were performed using six replicate plants of each treatment. **(A**–**C)** Mock inoculated plants; **(D**–**F)** plants inoculated with *Gluconacetobacter diazotrophicus* Pal5; **(G**–**I)** plants inoculated with *R. solanacearum* A21; **(J**–**L)** plants inoculated with *G. diazotrophicus* and with *R. solanacearum* A21.

**Figure 12:**
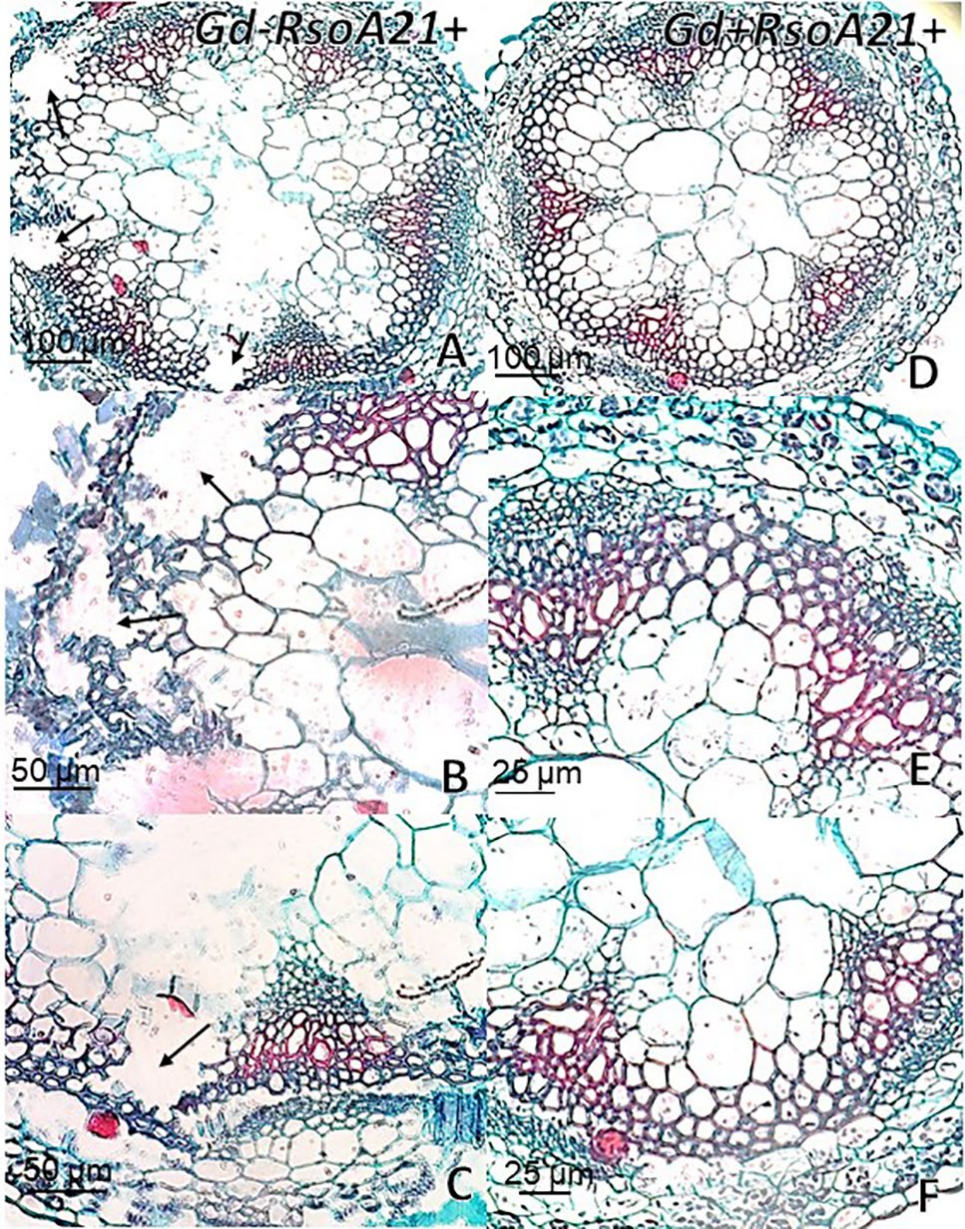
Arabidopsis thaliana plants inoculated 12 dpi with Ralstonia solanacearum A21. Stem cross sections dyed with safranin-fast green. **(A)** Plants inoculated with R. solanacearum A21; **(B**, **C)** are panel **(A)** detail of vascular bundles and areas of the stem cortical parenchyma. The black arrows indicate areas of damaged tissue. **(D)** Plants inoculated with Gluconacetobacter diazotrophicus Pal 5 and subsequently inoculated with R. solanacearum A21. **(E**, **F)** are panel **(D)** detail of vascular bundles and areas of the stem cortical parenchyma. Each image is a representative result of observation of at least 10 section from five biological replicates.

**Figure 13:**
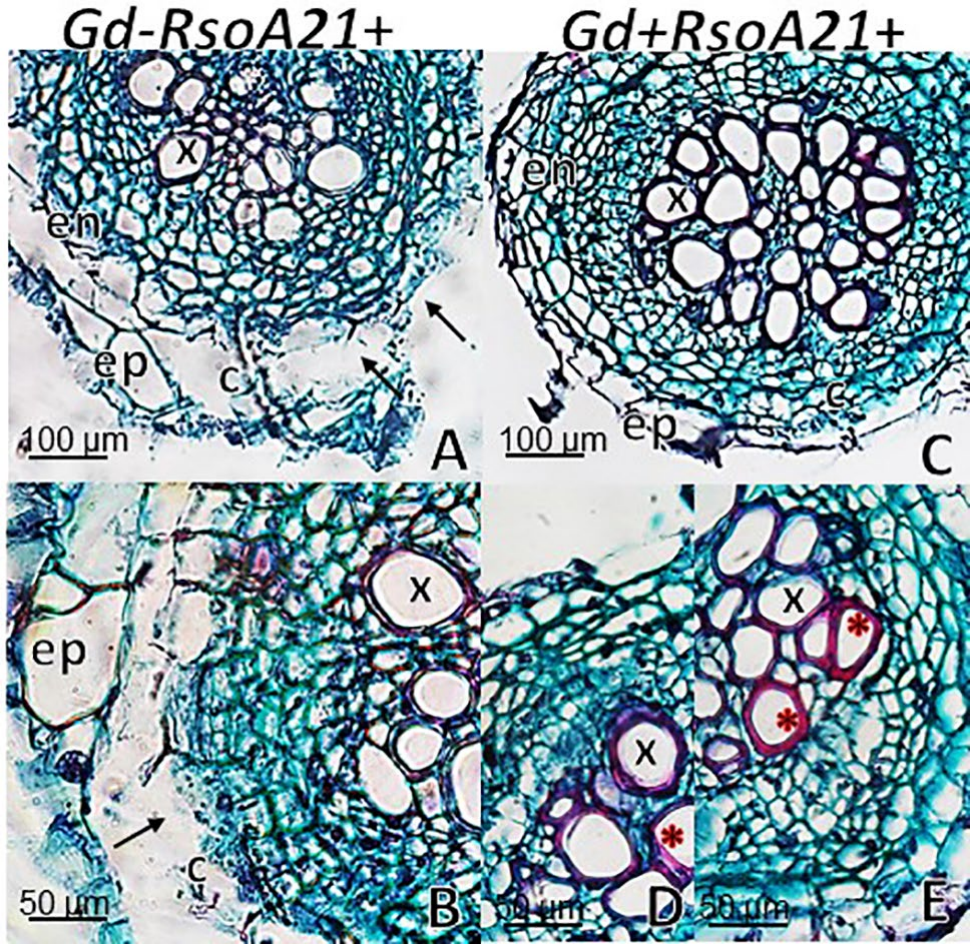
Arabidopsis thaliana plants inoculated 12 dpi with Ralstonia solanacearum A21. Root cross sections dyed with safranin-fast green. **(A)** Plants inoculated with R. solanacearum A21; **(B)** is panel **(A)** detail showing the epidermis, cortical zone, endodermis, and part of the vascular cylinder of the root. The black arrows indicate areas of damaged tissue. **(C)** Plants inoculated with Gluconacetobacter diazotrophicus Pal 5 and subsequently inoculated with R. solanacearum A21. **(D**, **E)** are panel **(C)** detail of xylem vessels of the vascular cylinder. The red asterisks show the highest lignification of the xylem vessels compared to panel **(B)**. c, cortical zone; en, endodermis; ep, epidermis; x, xylem vessel. Each image is a representative result of observation of at least 10 section from five biological replicates.

At 12 dpi, exomorphological changes began to be observed in plants inoculated only with *R. solanacearum* A21. The leaves of the rosette were chlorotic and dehydrated (**Figure 11I**). Stem and root cross sections showed significant anatomical changes at 12 dpi between the plants that were previously inoculated with *G. diazotrophicus* and those that did not possess the endophyte bacterium. Similarly, an increase in the amount of xylematic tissue, with vessels that present greater lignification and greater amount of sclerosed cortical parenchyma tissue between the vascular bundles were observed in those plants with *G. diazotrophicus* and *R. solanacearum* A21 (**Figures 12D–F**) as happened with the pathogenic strain *R. pseudosolanacearum* GMI1000 (data not shown). This was already observed to a lesser extent in plants only inoculated with *G. diazotrophicus* at 28 dpi (**Figures 2G, H**). These structural differences result in colonization resistance of the stems to the pathogenic bacteria. Seedlings treated with *Gd* ^+^ *RsoA21* ^+^ at 12 dpi presented integrity in all the tissues of the stem (**Figure 12D**) while seedlings treated with *Gd* ^−^ *RsoA21* ^+^, showed breakage of the cortical parenchyma and areas of the vascular bundles (**Figures 12A–C**). *R. solanacearum* A21 provokes plasmolysis of epidermal, cortical, and endodermal root cells in those seedlings without *G. diazotrophicus* (**Figures 13A, B**). Plasmolysis zones appear in alignment with one of the xylem pole axes. Integrity of all root tissues was observed in plants previously inoculated with *G. diazotrophicus* (**Figure 13C**), in addition, an increase in lignification of xylematic vessels was observed (**Figures 13D, E**).

CFU counting assays were performed to evaluate bacterial number (CFU/g) of *G. diazotrophicus* (28 dpi) and *R. solanacearum* A21 (12 dpi) in *A. thaliana* plants under each treatment. Results are indicated in **Table 1**. In roots of *A. thaliana* plants with *G. diazotrophicus*, *R. solanacearum* A21 counting decreased from (1.40 ± 0.68) x10^7^ CFU/g to (1.92 ± 2.31) x10^5^ CFU/g. *R. solanacearum* A21 was not detected in stems that had the endophytic bacteria. In stems of *A. thaliana* plants without *G. diazotrophicus,* CFU/g of *R. solanacearum* A21 was (3.79 ± 4.82) x10^5^. These results indicate that the bacterial population of *R. solanacearum* A21 decreases in the presence of *G. diazotrophicus*, and therefore this microorganism exerts some mechanism of biocontrol on *G. diazotrophicus*.

**Figure 14:**
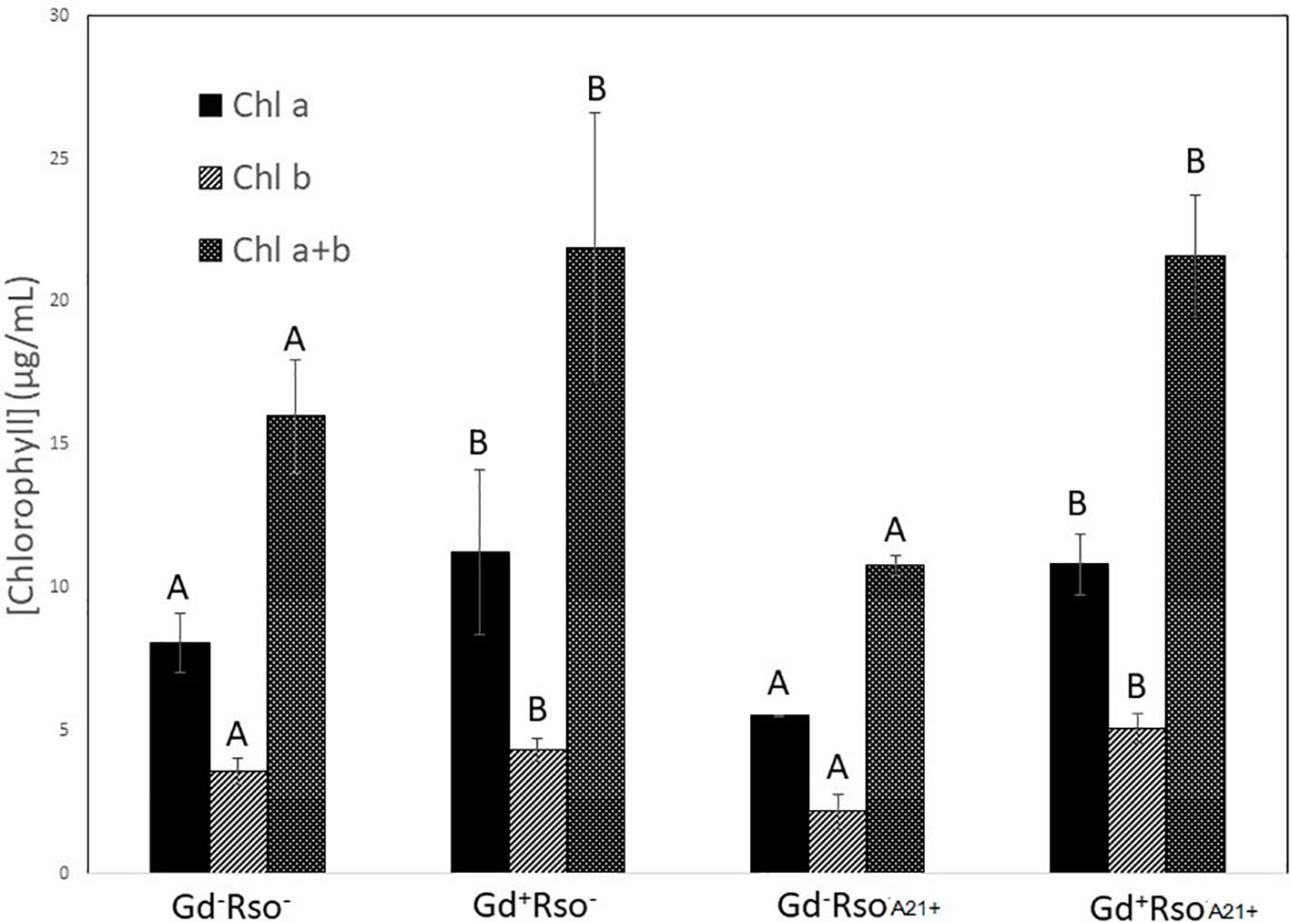
Bar graph showing the concentration of chlorophyll *a*, chlorophyll *b*, and chlorophyll *a + b* in leaves of *Arabidopsis thaliana* inoculated 28 dpi with *Gluconacetobacter diazotrophicus* (Gd^+^Rso^−^), inoculated 12 dpi with *Ralstonia solanacearum* A21 (Gd^−^RsoA21^+^), with both bacteria (Gd^+^ RsoA21^+^) or mock inoculated plants (Gd^−^Rso^−^). Concentration of pigments values are means of two leaves per plant of three plant replicates from each treatment. The experiments were performed independently by triplicate. The error bars represent the standard deviation. Significant differences between treatments are represented by different letters (ANOVA, *p* < 0.05).

Chlorophyll *a*, chlorophyll *b*, and chlorophyll *a + b* concentrations increased in those plants that were previously inoculated with *G. diazotrophicus* (Gd^+^ *RsoA21* ^−^ or Gd^+^ *RsoA21* ^+^). Plants only inoculated with *R. solanacearum* A21 presented lower concentrations of pigments. This treatment (Gd^−^ *RsoA21* ^+^) did not show significant statistical differences with the mock inoculated plants (**Figure 14**). The argentine *R. solanacearum* strain presented a deferential phenotype compared to GMI 1000 strain.

## Discussion

Plants live in complex environments where they interact closely with a wide range of microorganisms (Verhage et al., 2010). *G. diazotrophicus* is an endophytic bacterium able to colonize many plant species, both monocotyledons and dicotyledons. This bacterium can promote plant growth through different mechanisms that include the biological fixation of nitrogen, the secretion of phytohormones, the solubilization of mineral nutrients, and antagonism toward phytopathogens (Eskin et al., 2014).

During the last decades, several studies have tried to identify genes and regulatory proteins involved in the plant-*G diazotrophicus* association (dos Santos et al., 2010; Lery et al., 2011; Galisa et al., 2012; Bertini et al., 2014). However, many of the crops of agronomic interest are slow-growing species with a complex genome, and often their large size hinder their growth in greenhouses under controlled conditions, which limits the detailed molecular characterization of the plant-bacteria interaction. *A. thaliana* has a short life cycle (6 weeks) but has the typical characteristics of the other angiosperms in terms of morphology, anatomy, growth, development, and responses to the environment. Therefore, results can be obtained in a shorter time. *A. thaliana* is susceptible to only a limited number of pathogens, including viruses, bacteria, fungi, nematodes, and insects, and responds to pathogen attack similarly to species of higher plants (Andargie and Li, 2016). As a result, *A. thaliana* seedlings can be easily manipulated to study plant-endophyte interactions.

*R. solanacearum* is the bacterium responsible for the bacterial wilt of tomato and brown rot of potato (Agrios, 2005; Hayward, 1991). This bacterium is of great importance worldwide due to the enormous economic losses it causes, given that it infects a wide variety of crops and wild plants (Jones et al., 1991). The present study aimed to obtain *A. thaliana* seedlings with *G. diazotrophicus* and evaluate the protective potential of the endophytic bacterium against two phytophatogenic strains; *R. pseudosolanacearum* GMI1000 and *R. solanacearum* A21.

Our results show that the endophytic population of *G. diazotrophicus* was (2.57 ± 0.21) x10^5^ CFU/g in roots of *A. thaliana* seedlings, while for the stem the value was (2.60 ± 3.66) x10^5^ CFU/g at 28 dpi. The endophytic bacterial population of *G. diazotrophicus* in roots and leaves of *A. thaliana* was previously analyzed by Rangel de Souza et al. (2015) at different times after inoculation. They observed 1.5x10^6^, 3.1x10^6^, and 2.1x10^5^ CFU/g of root at 14, 28, and 50 dpi, respectively. They did not detect bacteria in the leaf at these times post-inoculation. The endophytic nature of *G. diazotrophicus* was confirmed in Brazil by counting this bacterium in roots, stems, and aerial parts of sugarcane (Reis et al., 1994). Values in all parts of the plant were between 10^3^ and 10^6^ CFU/g fresh weight. High values (10^6^–10^7^ CFU/g of fresh weight) were also found in sugarcane plants in India (Muthukumarasamy et al., 1999). There are other studies on sorghum, wheat, and tomato species where the bacterial population of *G. diazotrophicus* remains highest during the first days of infection (around seven or more) and then decreases and remains constant (Luna et al., 2012). Faced with these observations, Rangel de Souza et al. (2015) postulated that as the plant-endophyte interaction progresses, the bacterial population reduces, probably as a consequence of the plant’s defense system.

Rangel de Souza et al. (2015) reported inhibition in the growth of *A. thaliana* col-0 seedlings inoculated with *G. diazotrophicus* at 28 dpi. Unlike this, our observations did not register significant statistical differences in the growth parameters analyzed between the plants inoculated with *G. diazotrophicus* and the mock inoculated plants at 28 dpi (**Supplementary Figure 1**). Instead, an increase in xylematic tissue and a greater amount of sclerosed tissue were observed in the stems and roots of the inoculated plants compared to the mock-inoculated plants. Greater lignification was also observed in the xylem vessels of the inoculated plants (**Figure 2**). These anatomical changes induced by *G. diazotrophicus* in *A. thaliana* are part of the defense response of the plant primed by this bacterium. In addition, in this study a greater chloroplasts size was observed in the plants inoculated with *G. diazotrophicus* regarding to the mock inoculated plants at 28 dpi. Likewise, a significant difference was observed in the content of chlorophyll *a*, chlorophyll *b*, and chlorophyll *a+b* between plants inoculated with *G. diazotrophicus* and mock inoculated plants. Inoculated seedlings had a higher content of the pigments (**Figure 4**).

If a satisfactory symbiotic relationship is established with the endophyte, the biological fixation of nitrogen by the microorganism can supply a considerable part of the requirement of this nutrient. Nitrogen is essential for the synthesis of the Rubisco enzyme and for the synthesis of the light-harvesting complex which is strongly associated with chlorophyll molecules. About 70% of the nitrogen in the leaves exists in the chloroplasts and is mostly used to synthesize the photosynthetic machinery (Zhou et al., 2006). The biological fixation of nitrogen could stimulate the rate of photosynthesis through the increase of Rubisco activity and the speed of the photosynthetic electron transport chains (Harley et al., 1992). It is known that the microorganisms associated with plants stimulate photosynthesis because they use photosynthates as a carbon source for their growth, diverting them from their real destiny in the plant, which is why they are forced to increase the rate of photosynthesis to supply their requirements (Kaschuk et al., 2009). As occurs in soybean plants (Abu-Shakra et al., 1978), it could happen that the synergistic effect of the prolonged acquisition of nitrogen, and the stimulation of photosynthesis by these microorganisms, postpone the degradation of the proteins present in the leaves and also of the chlorophyll. This could explain the observed increase of photosynthetic pigments in plants inoculated with *G. diazotrophicus* without an increase in the size of the plants at 28 dpi.

It is known that most phytopathogenic microorganisms have biological antagonists that can be used for biological control. In recent years, the use of antagonistic bacteria and fungi in agricultural diseases treatment has become more relevant (Robles Carrión, 2012). In this work the behavior of the phytopathogenic strains of *R. solanacearum* and the endophyte bacteria *G. diazotrophicus* in the plant was analyzed in order to gain knowledge about benefits of *G. diazotrophicus* against the pathogen causing disease in solanaceous crops. For this, plants that were previously inoculated with *G. diazotrophicus* and in which the endophytic bacteria had already been established, were subsequently infected with the strains GMI1000 or A21 of *R. solanacearum*. Symptoms of chlorosis and dehydration began to be observed at 12 dpi in plants treated with *R. solanacearum* (GMI1000 or A21) (**Figures 5I** and **11I**). Symptoms were more pronounced when the strain used was GMI1000. Chlorophyll content of plants treated with *R. pseudosolanacearum* GMI1000 was the lowest regarding the other treatments (**Figure 10**); whereas for plants treated with *R. solanacearum* A21 it did not show statistical differences with the chlorophyll content of mock inoculated plants (**Figure 14**). These results would indicate that the A21 strain of *R. solanacearum* presents a different phenotype from that of the GMI1000 strain and therefore the responses of the *A. thaliana* plants to the infection is also different. Exomorphological signs observed in *A. thaliana* seedlings treated with GMI1000 or A21 strains of *R. solanacearum* were compatible with the symptoms caused by this phytopathogen in the *A. thaliana* species (Deslandes et al., 1998). Plants previously inoculated with *G. diazotrophicus* and infected later with GMI1000 or A21 strains of *R. solanacearum* did not show symptoms of disease (**Figures 5L** and **11L**).

Although between 6 and 9 dpi the phytopathogenic bacterium does not generate changes in the exomorphological aspect of the plants in any of the treatments, presence of bacteria could be detected along the stem in samples stained with toluidine blue in plants of *A. thaliana* treated only with *R. pseudosolanacearum* GMI1000 (**Supplementary Figures 2A–H**). Presence of a strain GMI1000-GFP was confirmed in roots and stems of *A. thaliana* seedling treated only with this strain by confocal microscopy (**Figures 6A, B** and **7A, B**).

The interaction of *G. diazotrophicus* Pal5 and *R. solanacearum* A21 and its anatomical effects on *A. thaliana* plants were analyzed. Stem cross sections stained with safranin-fast green of *A. thaliana* seedlings treated with endophytic and pathogenic bacteria showed tissue integrity with greater lignification of xylematic vessels and sclerosed cortical parenchyma between the vascular bundles (**Figures 12D, E**). The opposite was observed for plants only inoculated with *R. solanacearum* A21 (**Figures 12A–C**). The structural differences allowed the stems to conserve their structure better than those plants infected only with pathogenic bacterium. In this case, *A. thaliana* seedling with *G. diazotrophicus* elicited a resistance mechanism to *R. solanacearum* A21 infection. Roots of *A. thaliana* plants without *G. diazotrophicus* were more easily colonized as observed in **Figure 13**. Plasmolized epidermal, cortical, and endodermal root cells were evidenced according to Digonnet et al. (2012), who described the route of *R. solanacearum* colonization in *A. thaliana* roots. Greater lignification of xylematic elements of vascular cylinder and mayor integrity of cortical and endodermal root cells were observed in roots of plants with *G. diazotrophicus* (**Figures 13D, E**).

Bacterial counting assays were also performed to determine microbial populations resulting from the interaction of endophytic and phytopathogenic bacteria. Both the microbial population of *R. pseudosolanacearum* GMI1000 and *R. solanacearum* A21 decrease in roots of *A. thaliana* seedlings in the presence of *G. diazotrophicus*. Stem extracts and dilutions of *A. thaliana* seedlings treated with *G. diazotrophicus* and *R. solanacearum* (GMI1000 or A21) did not show growth in the selective medium mSMSA for pathogenic bacterium (**Table 1**).

This and previous results indicated that the bacterial population of *R. solanacearum* A21 and *R. pseudosolanacearum* GMI1000 decreased in the presence of *G. diazotrophicus* in *A. thaliana* seedlings, therefore this endophyte microorganism would be exerting a mechanism of biocontrol on the phytopathogen.

Biocontrol mechanisms by beneficial microorganisms include: i) direct interference with pathogens, this may be through competition for nutrients and space, the secretion of antibiotics, or the degradation of virulence factors; ii) the induction of resistance by the host plant, which is often related to the induced systemic resistance (ISR) that involves ET and JA phytohormones (Haas and Défago, 2005; Van Wees et al., 2008). In the case of beneficial microorganisms, ISR is usually associated with priming the defense routes for an enhanced response, rather than directly activating of the defense system (Van Wees et al., 2008; Zamioudis and Pieterse, 2012). Filgueiras et al. (2019) observed an increase in PR-10 a pathogenesis related protein, a marker of the JA/ET defense route in rice plants when they were inoculated with *G. diazotrophicus*. This endophyte activated a similar response in sugarcane plants since these plants inoculated with *G. diazotrophicus* were more resistant to infection with pathogens such as *X. albilineans*, *C. falcatum*, and *Meloidogyne incognita*. De Nogueira et al. (2001) and Cavalcante et al. (2007) demonstrated the activation of genes involved in the ET signaling pathway in sugarcane plants colonized by *G. diazotrophicus*. Dong et al. (1995; 1997) reported the accumulation of polysaccharides and tannins in the parenchymal cells surrounding the metaxylem vessels of sugarcane plants inoculated with *G. diazotrophicus* suggesting that the plant’s defense system is activated during the interaction with the bacteria. The increased lignification in xylem elements and sclerosis of diverse tissue in both stems and roots of *A. thaliana* col-0 inoculated with *G. diazotrophicus* are concordant with Dong et al. (1997) observations. In the present work confocal microscopy technique was used and *R. pseudosolanacearum* GMI1000 with GFP to observe the presence of this bacterium in roots and stems of *A. thaliana* Col 0. Roots of *A. thaliana* plants treated with *G. diazotrophicus* and *R. pseudosolanacearum* GMI1000-GFP showed the phytopathogenic bacteria arrested in the cells surrounding the metaxylematic vessels, without colonizing them (**Figures 7C–D**). This could be due to antibacterial compounds present in these cells or to cell wall modifications of the metaxylematic vessels. Nakaho et al. (2000) reported thicker electron-dense pit membranes in resistant tomato cultivars resulting in a limited movement of *R. solanacearum*. Meanwhile, Caldwell et al. (2017) proposed that the differential colonization of *R. solanacearum* in resistant and susceptible tomato roots was due to the ability of the resistant cultivars, through different mechanisms, to restrict bacterial root colonization in time and space. Therefore, xylem vessel structure could determine the plant response to this phytopathogen. On the other hand, Rangel de Souza et al. (2015) reported the participation of the defense mechanism mediated by SA when inoculated *A. thaliana* Col-0 with *G. diazotrophicus*. *NahG* mutant plants, which present a bacterial salicylate hydroxylase that degrades SA, showed no growth problems during the first stages of infection with *G. diazotrophicus* as did Col-0 plants of *A. thaliana*. Conn et al. (2008) reported that an endophytic actinobacteria in plants of *A. thaliana* Col-0, is able to priming for both defense routes, the SAR route, and the JA/ET route, regulating “upstream” genes in both pathways depending on the pathogen that later infects the plant. So, the resistance to the bacterial pathogen *Erwinia carotovora* subsp. *carotovora* required the JA/ET route and, on the other hand, the resistance to the fungal pathogen *Fusarium oxysporum* involved the SAR response.

Plants presented many specialized defense mechanisms; the plant cell wall represents a fundamental line of defense. The cell wall is reinforced with a complex structure, so-called papillae, at sites of interaction with foreign microorganisms. Papillae are formed between the plasma membrane and the cell wall. The biochemical composition of papillae may vary between plant species, but some commonly compounds include reactive oxygen species, phenolics, cell wall proteins, and polymers such as (1,3)-β-glucan callose (Voigt, 2014; Ellinger and Voigt, 2014). Ellinger et al. (2013) reported that timing of the different papilla-forming transport processes is important factor to slow or even stop pathogen invasion. In the present work, *G. diazotrophicus* prime the cellular defense response that involves the deposition of callose. The formation of a discrete number of small papillae in root hairs of *A. thaliana* plants treated with *G. diazotrophicus* was observed, indicating that this is the entry site chosen by this endophyte (**Figures 8 D–F**). The formation of papillae in plants treated with *R. pseudosolanacearum* GMI1000 was observed both in cells of the root surface (**Figures 8G–I**) and in the root hairs (**Figures 8G, H**). In plants previously treated with *G. diazotrophicus* and then with *R. pseudosolanacearum* GMI1000, the papillae were larger and more abundant, indicating a rapid response to their formation (**Figures 8J–L**). Similarly, callose deposition in trichomes and leaf tissue of *A. thaliana* Col 0, was greater in those plants that had previously been inoculated with the bacterial endophyte and then were infected with *R. pseudosolanacearum* GMI1000 (**Figures 9G, H**). The participation of the phytohormone SA in the biocontrol by *G. diazotrophicus* of *R. solanacearum* was confirmed using a *sid2* mutant of *A. thaliana* in the SA biosynthesis. In these plants no callose deposition on the development of papilla was observed in radical hairs of the roots (see **Supplementary Figure 6**). In addition the absence of callose was also evident in trichomes and areas of the epidermis in the *sid2* mutants with all treatments (**Supplementary Figure 5**). Ahn et al. (2007) demonstrated that a non-pathogenic rhizobacteria, *Pseudomonas putida* LSW17S allowed a strong and rapid transcription of defense genes and the accumulation of hydrogen peroxide and callose in plants of *A. thaliana* Col 0 infected with *Pseudomonas syringae* pv. *tomato* DC3000. LSW17S prime the resistance to the disease in *A. thaliana* plants *via* the activation of SAR and JA/ET routes.

Endophytic bacterium produces a protective effect in the plant against the pathogen through the cell wall reinforcement and the increase in lignin and callose, preventing the successful colonization of the pathogenic bacteria which is evidenced by a smaller amount of bacteria in the plant and a delayed phenotype of the appearance of damage to plant tissue and chlorosis caused by the phytopathogenic bacteria. All these modifications lead to an increase in the plant defense response and are extremely linked with the production of SA. Altogether these results indicate that *G. diazotrophicus* colonizes *A. thaliana* plants through the radical hairs, inducing the resistance to *R. solanacearum* infection by mechanism such as papillae formation that contains callose and structural changes in xylem vessels

Our study also provides results about a new typified strain of *R. solanacearum*, A21, isolated from tomato crops of Argentinean northeast region. *G. diazotrophicus* also prevents the advance of the infection of this strain of *R. solanacearum*. Our work opens new insight in the integrated management of production/protection of intensive agronomic crops of interest attacked by *R. solanacearum*.

## Data Availability Statement

The raw data supporting the conclusions of this manuscript will be made available by the authors, without undue reservation, to any qualified researcher.

## Author Contributions

MR and EO conceived, designed and direct the work. MR and NA carried out the experiments, performed the measurements and analyzed the data. JT and AC contributed in carried out and planned the biocontrol assays. MSS contributed in carried out the colonization assays. MIS and VF performed molecular typing of Argentinean isolated strains. MM and AAC contribute to histological evaluations. All authors contributed to analysis and interpretation of results. MR, MIS and EO wrote the manuscript. JT, AC and MSS revised and corrected the manuscript. All authors have made substantial, direct and intellectual contribution to the work. All the authors have read the final manuscript and approved the submission.

## Funding

This work was supported by the Agencia Nacional de Promoción Científica y Tecnológica (ANPCyT PICT 2017-2242 to EO) and by Science and technology Secretary from Rosario National University (UNR) [Grant BIO432] to EO.

The funders had no role in study design, data collection and analysis, decision to publish, or preparation of the manuscript.

## Conflict of Interest

The authors declare that the research was conducted in the absence of any commercial or financial relationships that could be construed as a potential conflict of interest.
