# Supplementary material for: Anatomical and Biochemical Changes Induced by Gluconacetobacter diazotrophicus Stand Up for Arabidopsis thaliana Seedlings From Ralstonia solanacearum Infection
Source: Front Plant Sci. 2019 Dec 23;10:1618. doi: 10.3389/fpls.2019.01618 (PMC6936193; doi:10.3389/fpls.2019.01618)
Supplement: Supplementary file 1 [file DataSheet_1.docx]

Supplementary Material

# Supplementary Data

# Not applicable

# Supplementary Figures and Tables

## SupplementaryTables

**Supplementary table 1.** Bacterial strains and culture media used in this work

| Bacterial strain^a^ | Description | Reference^b^ |
| --- | --- | --- |
| *Gd* | *Gluconacetobacter diazotrophicus*, endophyte wild type strain |  |
| *Rpso*GMI1000 | *Ralstonia pseudosolanacearum* GMI1000Wild type strain, Phylotipe I, Origin: French Guyana. | Salanoubat et al., 2002; Safni et al., 2018; Prior et al., 2016. |
| *Rpso*GMI1000-GFP | *Ralstonia pseudosolanacearum* GMI1000-GFP,*Pep::GFP*, Gm^R^ | Monteiro et al., 2012b |
| *Rso* A21 | *Ralstonia solanacearum* ArgentineIsolation of tomato, variety “El pida” (Bella Vista, Corrientes) | Thiswork |
| Growth medium | **Composition** | **Reference** |
| BG (Bacto-Glucose) | 10 g/L casein peptone, 1 g/L yeast extract, 1 g/L casaminoacids | Clough et al., 1994 |
| LGI-P  Selective growth medium | 100 g/Lsucrose, 0.2 g/L K_2_HPO_4_, 0.6 g/L KH_2_PO_4_, 0.2 g/L MgSO_4_·7H_2_O, 0.2 g/L CaCl_2_·2H_2_O, 0.002 g/L Na_2_MoO_4_·H_2_O, 0.01 g/L FeCl_3_·6H_2_O, pH5.5 |  |
| mSMSA | 1 g/L casaminoacids, 10 g/L peptone, 5 g/L glucose | Englebrecht et al., 1994 modified by Elphinstone et al., 1996 |

^a^Abbreviations: *Gd*: Gluconacetobacter diazotrophicus; *Rpso*GMI1000: *R. pseudosolanacearum* GMI1000; *Rpso*GMI1000-GFP: *R. pseudosolanacearum* GMI1000-GFP; *Rso*A21: *R. solanacearum*

^b^References:

Clough, S., Schell, M. A., Denny, T. (1994). Evidence for involvement of a volatile extracellular factor in Pseudomonas solanacearum virulence gene expression. *MolecularPlant*-*Microbe* Interactions. <https://doi.org/10.1094/MPMI-7-0621>.

Elphinstone, J. G., Hennessy, J.,Wilson, J. K., Stead, D. E. (1996). Sensitivity of different methods for the detection of *Ralstoniasolanacearum* in potato tuber extracts. EPPO Bull. 26, 663–678. doi: 10.1111/j.1365-2338.1996.tb01511.x.

Englebrecht. M.C. (1994) Modification of a semi-selective medium for the isolation and quantification of *Pseudomonassolanacearum*. In Bacterial Wilt Newsletter (ed. Hayward, A. C.) 10. 3-5. Australian Centre for International Agricultural Research, Canberra (AU).

Monteiro F., Solé, M., van Dijk, I.., Valls, M. (2012b) A chromosomal insertion toolbox for promoter probing, mutant complementation, and pathogenicity studies in *Ralstoniasolanacearum*. *Mol Plant Microbe Interact.*25, 557-568. doi: 10.1094/MPMI-07-11-0201.

Prior, F., Ailloud, Dalsing, B. L.,Remenant, B.,Sanchez,B., Allen, C. (2016) “Genomic and proteomic evidence supporting the division of the plant pathogen *Ralstoniasolanacearum* into three species”. *BMC Genomic 17: 90*. doi: 10.1186/s12864-016-2413-z..

Safni, I., Cleenwerck, I., De Vos, P., Fegan, M., Sly, L., Kappler, U. (2014) “Polyphasic taxonomic revision of the *Ralstonia solanacearum* species complex: proposal to emend the descriptions of *Ralstoniasolanacearum* and *Ralstoniasyzygii* and reclassify current *R . syzygii* strains as *Ralstonia syzygii* subsp . *syzygii* subsp . nov., *R. solanacearum* phylotype IV strains as *Ralstonia syzygii* subsp . *indonesiensis* subsp . nov., banana blood disease bacterium strains as *Ralstonia syzygii* subsp. *celebesensis* subsp. nov. and *R. solanacearum* phylotype I and III strains as *Ralstonia pseudosolanacearum* sp. nov”. *Int. J. Syst. Evol. Microbiol*. 64, 3087–3103.

Salanoubat, M., Genin, S., Artiguenave, F., Gouzy, J., Mangenot, S., Arlat, M., Billault, A., Brottier, P., Camus, J. C., Cattolico, L., Chandler, M., Choisne, N., Claudel-Renard, C., Cunnac, S., Demange, N., Gaspin, C., Lavie, M., Moisan, A., Robert, C., Saurin, W., Schiex, T., Siguier, P., Thébault, P., Whalen, M., Wincker, P., Levy, M., Weissenbach, J, Boucher, CA. “Genome sequence of the plant pathogen *Ralstoniasolanacearum*” Nature 415, 497–502.

## SupplementaryFigures


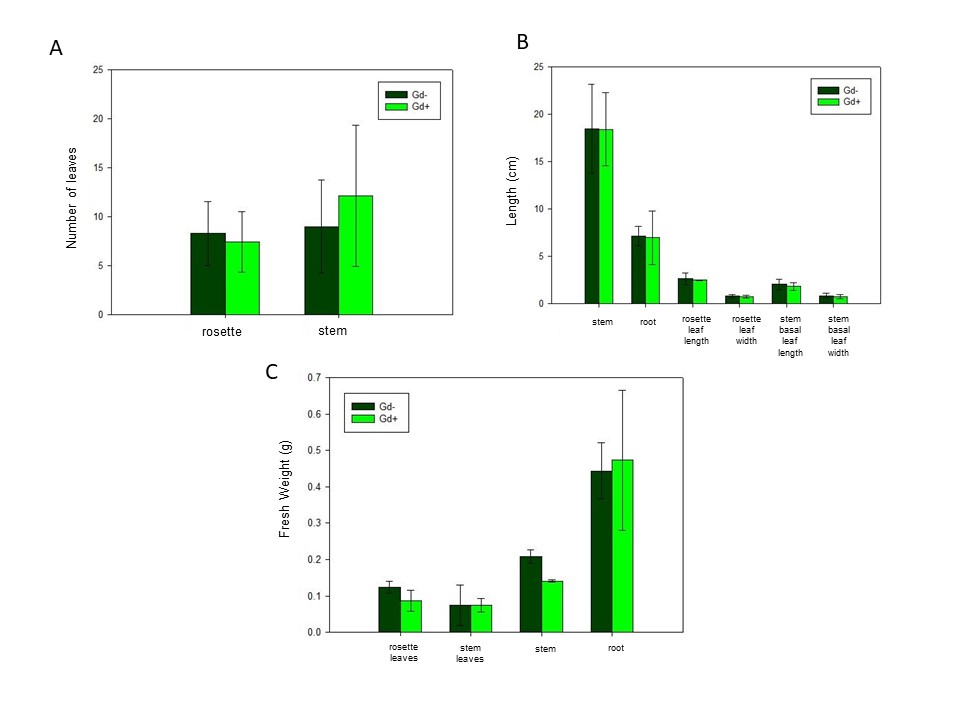
**Supplementary Figure 1.** Bar graph showing different growth parameters in *A. thaliana* col 0 plants inoculated with *G. diazotrophicus* (Gd+) and mock inoculated plants (Gd-). (A) number of rosette leaves, number of stem leaves; (B) length of the main root, length of the stem; (C) fresh weight of the different organs: rosette leaves, stem leaves, stem and root, size of rosette leaves, size of stem basal leaves.The error bars represent the standard deviation. Significant differences between treatments are indicated by an asterisk (Student's t test, *p*<0.05).


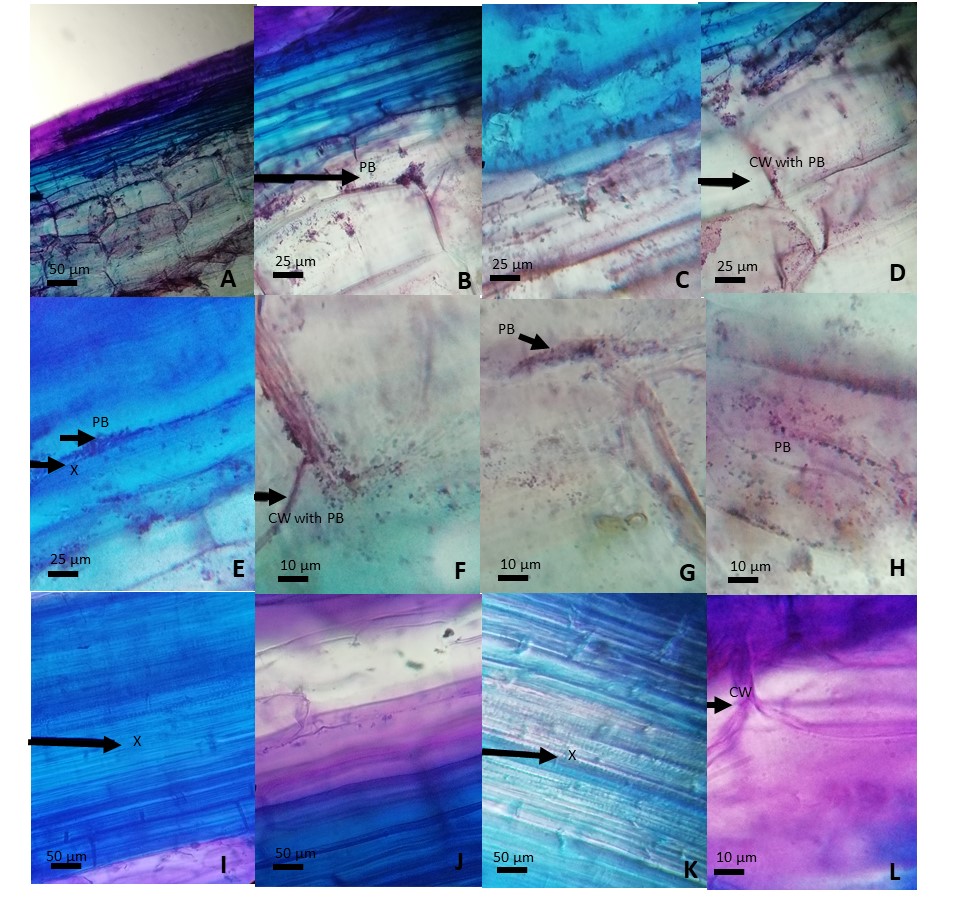


**Supplementary Figure 2. A-H**, Plants of *A. thaliana* Col 0 inoculated with *R. pseudosolanacearum* GMI1000 6 dpi. **I-L**, Plants of *A. thaliana* inoculated with *G. diazotrophicus* 22 dpi and *R. solanacearum* GMI1000 6 dpi. **A-L**, longitudinal section of stems stained with 1% (w/v) toluidine blue.CW: cell wall; X: xylem; PB: phytopathogenic bacteria.


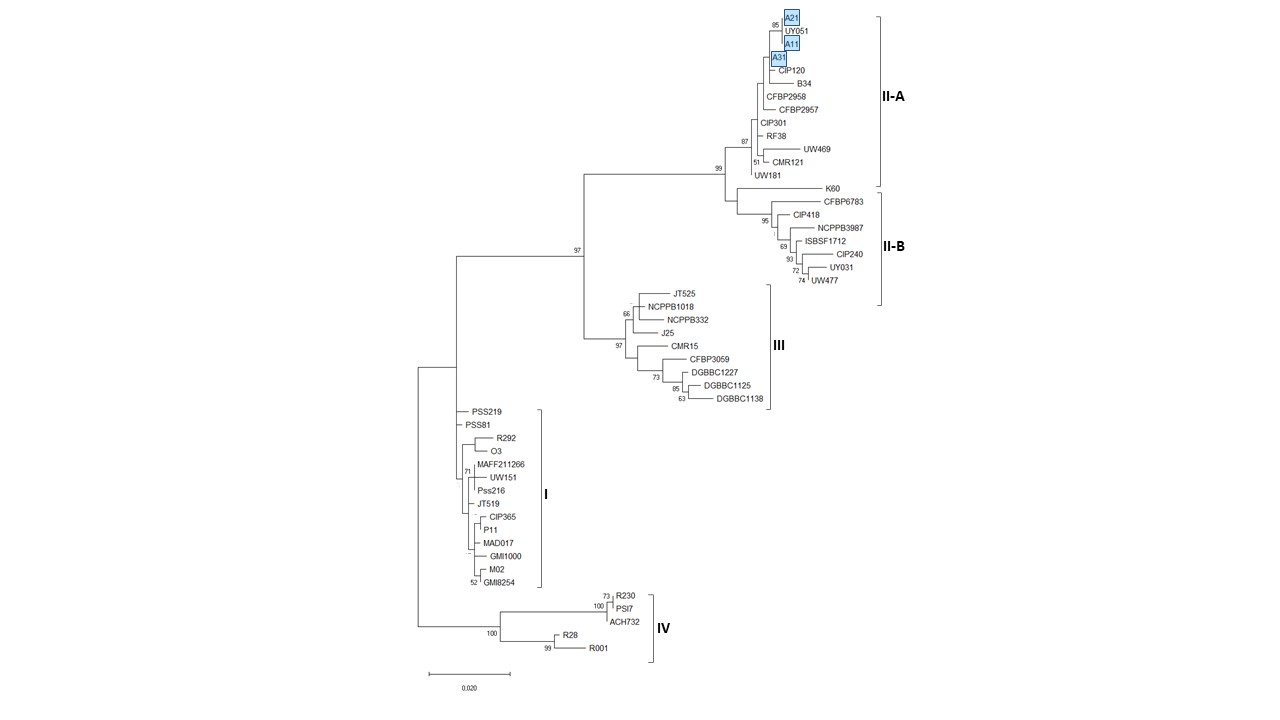


**Supplementary Figure 3.** Phylogenetic tree based on partial *egl*sequences of 3 *Ralstonia solanacearum* strains isolated in Argentina (A11, A21, A31) and 45 strains belonging to the phylotypes I-IV within the *Ralstonia solanacearum* species complex. The evolutionary history was inferred by using the Maximum Likelihood method and Tamura-Nei model (TamuraandNei, 1993). The tree with the highest log likelihood (-2430,81) is shown. Initial trees for the heuristic search were obtained automatically by applying Neighbor-Join and BioNJ algorithms to a matrix of pairwise distances estimated using the Maximum Composite Likelihood (MCL) approach, and then selecting the topology with superior log likelihood value. The tree is drawn to scale, with branch lengths measured in the number of substitutions per site. Bootstrap values higher than 50% are shown next to the branches. This analysis involved 49 nucleotide sequences. There were a total of 653 positions in the final dataset. Evolutionary analyses were conducted in MEGA X (Kumar et al. 2018).

References of Supplementary Figure 3:

1. Tamura K. and Nei M. (**1993**). Estimation of the number of nucleotide substitutions in the control region of mitochondrial DNA in humans and chimpanzees. *Molecular Biology and Evolution* **10**:512-526.

2. Kumar S., Stecher G., Li M., Knyaz C., and Tamura K. (**2018**). MEGA X: Molecular Evolutionary Genetics Analysis across computing platforms. *Molecular Biology and Evolution* **35**:1547-1549.


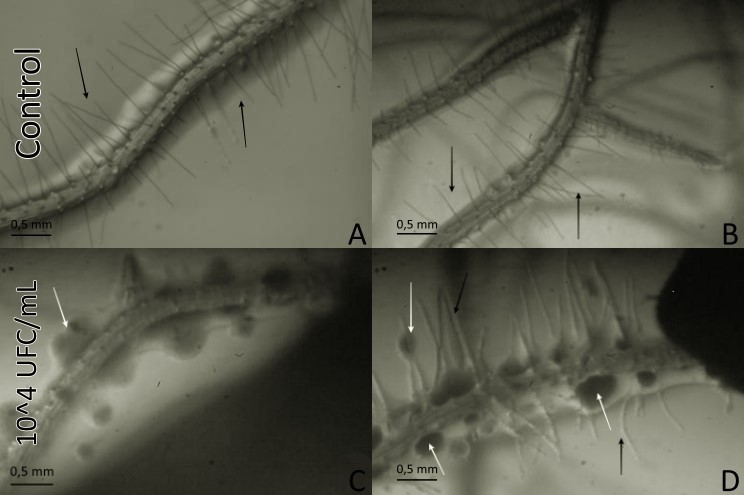


**Supplementary Figure 4**. Roots of *A. thaliana* Col 0 seedlings grown *in vitro* observed with a stereoscopic microscope at 9 dpi with *G. diazotrophicus* Pal5. A-B, control plants without inoculation; C-D, inoculated plants with suspension of *G. diazotrophicus* Pal5 with a concentration of 10^4^ CFU / mL. White arrows indicate bacterial colonies; Black arrows indicate radical hairs.


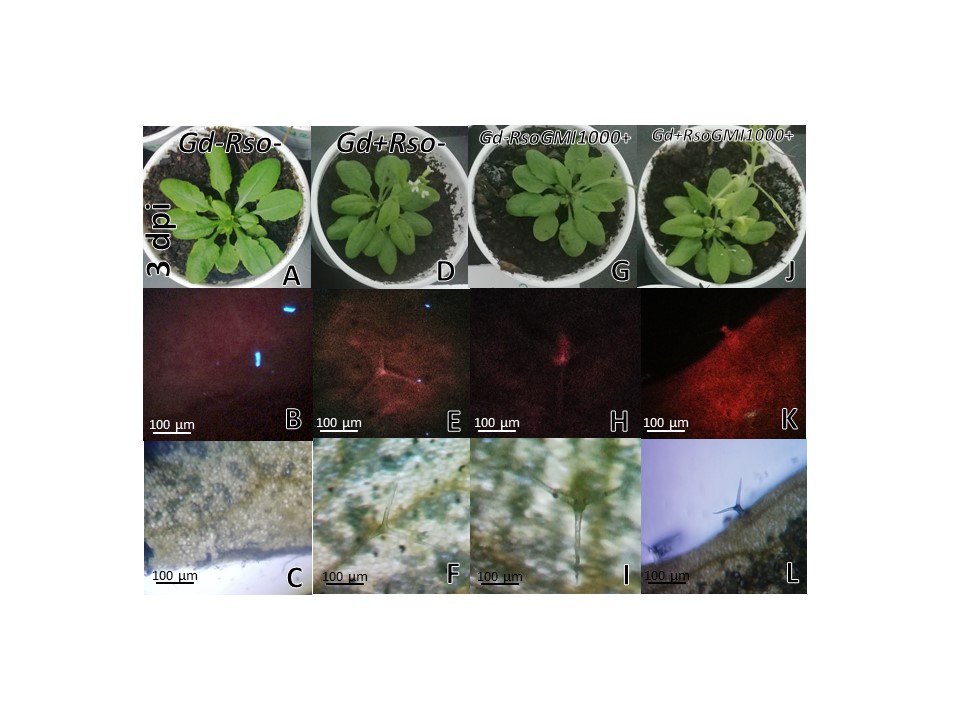


**Supplementary Figure 5.** *A. thaliana sid2* mutants grown with different treatments, images are shown at 3 days post-inoculation with *R. pseudosolanacearum* GMI1000. (A-C) mock inoculated plants; (D-F) plants inoculated with *G. diazotrophicus* Pal5; (G-I) plants inoculated with *R. pseudosolanacearum* GMI1000; (J-L) plants inoculated with *G. diazotrophicus* and with *R. pseudosolanacearum* GMI1000. B-K, epifluorescence microscope micrographs of *A. thaliana sid2* mutants leaves treated with aniline blue 0.05 % w/v showing portions of the epidermis and trichomes. C-L, light microscope micrographs of *A. thaliana sid2* mutant leaves treated with aniline blue 0.05 % w/v showing the same as in the pictures B-K.

**
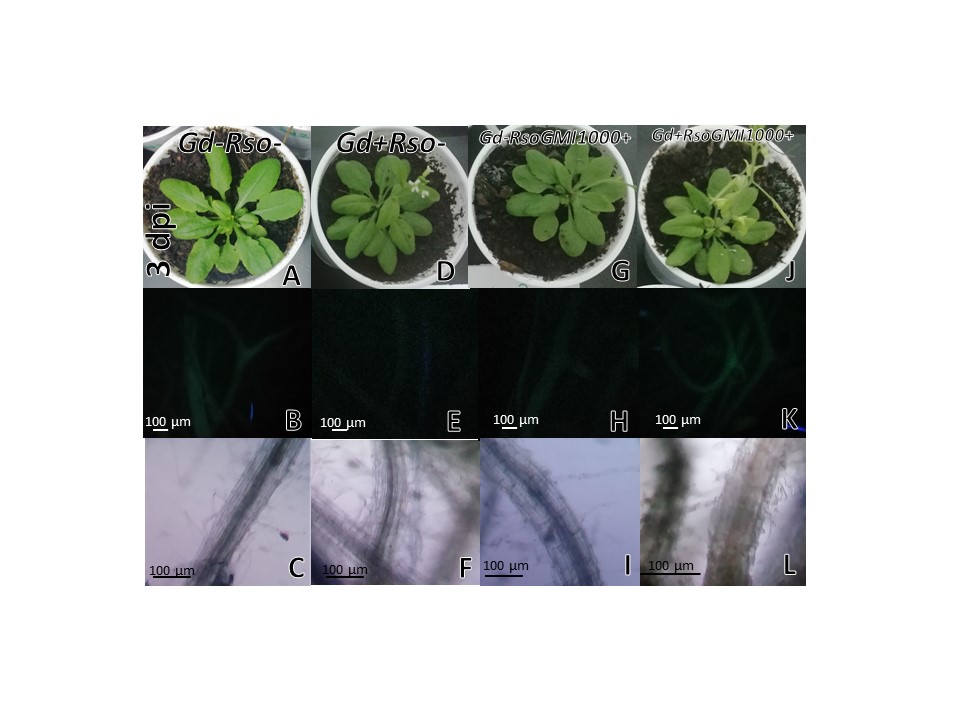
Supplementary Figure 6.** *A. thaliana sid2* mutants grown with different treatments, images are shown at 3 days post-inoculation with *R. pseudosolanacearum* GMI1000. (A-C) mock inoculated plants; (D-F) plants inoculated with *G. diazotrophicus* Pal5; (G-I) plants inoculated with *R. pseudosolanacearum* GMI1000; (J-L) plants inoculated with *G. diazotrophicus* and with *R. pseudosolanacearum* GMI1000. B-K, epifluorescence microscope micrographs of *A. thaliana sid2* mutant roots treated with aniline blue 0.05 % p/v. C-L, light microscope micrographs of *A. thaliana sid2* mutant roots treated with aniline blue 0.05 % p/v showing radical hairs. No deposits of callose are observed in the papillae of the radical hairs of any of the treatments tested (B-K).


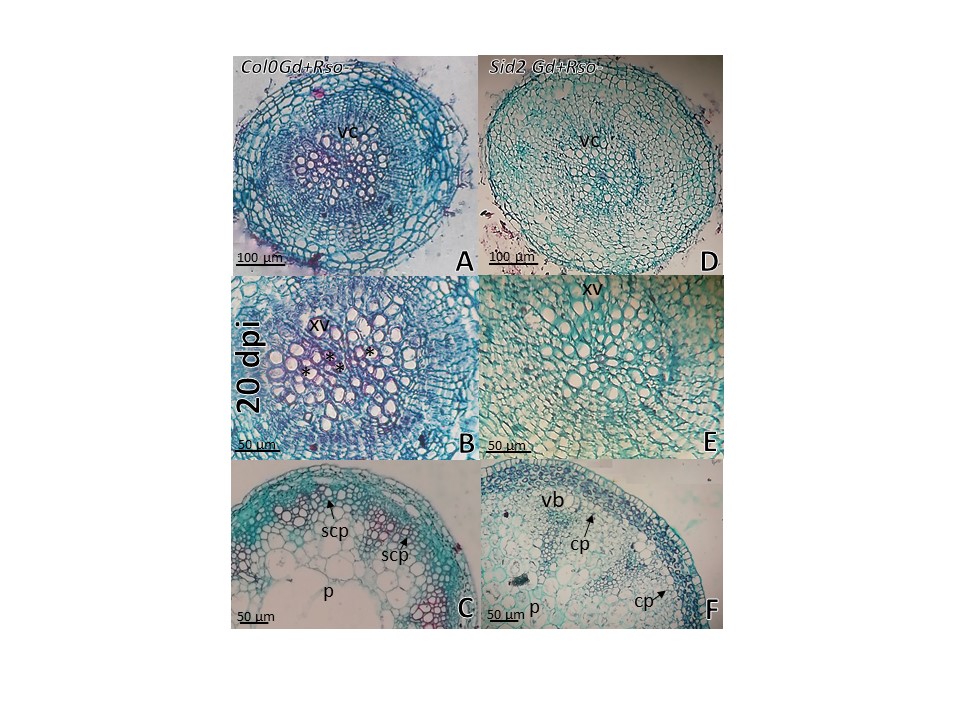


**Supplementary Figure 7.** Cross sections micrographs of *A. thaliana sid2* mutants and Col 0 plants inoculated with *G. diazotrophicus* at 20 dpi. (A, B-D, E) Root; (C,F) Stem. Staining with safranin-Fast Green. B, Root vascular cylinder details of *A. thaliana* Col 0(A) showing lignification of the xylematic vessels*.* E, Root vascular cylinder details of *A. thaliana sid2* mutants (D) showing low lignification of the xylematic vessels. C, Stem of *A. thaliana* col 0 plants showing a greater lignifications and amount of xylematic tissue and sclerosed cortical parenchyma than in *A. thaliana sid2* mutants (F). cp=cortical parenchyma, p=pith, scp=sclerosed cortical parenchyma, vb=vascular bundle, vascular cylinder, xv=xylematic vessels. Asterisk indicated more xylematic vessels lignification.
